# Heat wave Intensity Duration Frequency Curve: A Multivariate Approach for Hazard and Attribution Analysis

**DOI:** 10.1038/s41598-019-50643-w

**Published:** 2019-10-01

**Authors:** Omid Mazdiyasni, Mojtaba Sadegh, Felicia Chiang, Amir AghaKouchak

**Affiliations:** 1 Department of Civil and Environmental Engineering, University of California, Irvine, California, 92697 USA; 2 Department of Civil and Environmental Engineering, Boise State University, Idaho, 83725 USA; 3 Department of Earth System Science, University of California, Irvine, California, 92697 USA

**Keywords:** Attribution, Climate and Earth system modelling, Natural hazards

## Abstract

Atmospheric warming is projected to intensify heat wave events, as quantified by multiple descriptors, including intensity, duration, and frequency. While most studies investigate one feature at a time, heat wave characteristics are often interdependent and ignoring the relationships between them can lead to substantial biases in frequency (hazard) analyses. We propose a multivariate approach to construct heat wave intensity, duration, frequency (HIDF) curves, which enables the concurrent analysis of all heat wave properties. Here we show how HIDF curves can be used in various locations to quantitatively describe the likelihood of heat waves with different intensities and durations. We then employ HIDF curves to attribute changes in heat waves to anthropogenic warming by comparing GCM simulations with and without anthropogenic emissions. For example, in Los Angeles, CA, HIDF analysis shows that we can attribute the 21% increase in the likelihood of a four-day heat wave (temperature > 31 °C) to anthropogenic emissions.

## Introduction

Heat waves have significant negative implications on human health, urban air quality, ecological and environmental conditions, as well as agricultural and energy sectors^1–5^. In addition, heat waves have been connected to the increased risk of forest fires^6^. Heat waves are also considered to be one of the deadliest natural hazards, and cause high mortality rates in both developed and developing countries^7^. For example, the 2003 European heat wave and 2010 Russian heat wave killed over 70,000 and 56,000 people, respectively^8–12^. The 2003 European heat wave also caused electricity demand to soar and energy efficiency to plummet^13^. France, Europe’s main electricity exporter, was forced to cut power exports by more than half during the heat wave, because power plants were operating at significantly reduced capacity^14,15^. Extreme temperatures and heat wave events have also caused problems in the transportation sector. Phoenix Sky Harbor Airport was forced to cancel nearly 50 flights due to extreme temperatures in the summer of 2017, when temperatures soared as high as 120 °F. These cancellations produced a domino effect on the entire air transportation system, which demonstrate how impacts of regional heat waves can expand to a national or even global level.

Rising global temperatures are expected to increase the intensity, duration, and frequency of heat waves around the world^16–21^,^22–24^. Most studies investigate different features of heat waves independently, and ignore their relationships^25–28^. Although there is no universal definition, heat waves are typically described as a consecutive period of hot days with temperatures above a given threshold^7,29^. The threshold is often based on a percentile of each month’s daily temperatures or a fixed value^30–32^. For a comprehensive review of traditional heatwave definitions, and a toolbox to identify heatwaves and their statistics at a global scale refer to Raei, 2018^32^. Current metrics evaluate individual heat wave characteristics, such as the hottest day of each year or longest duration of consecutive hot days^7,17,22,29,31,33^. However, current univariate indicators often underestimate the impacts of heat waves because they fail to characterize the extreme event in a comprehensive manner^23,34,35^. The impacts of individual heat wave characteristics can be amplified when considered concurrently (e.g. high intensity and long duration vs high intensity and short duration heat wave events). The significant impacts along with the increasing intensity and duration of extreme heat wave events highlight the need for a comprehensive metric, accounting for all heat wave characteristics simultaneously.

In this paper, we propose a heat wave intensity-duration-frequency (HIDF) model. This model differs from the typical definition of heat waves, as we model the annual occurrence probability of consecutive hot days. We define HIDF to be similar to traditional precipitation IDF curves, obtaining the occurrence probability (or the corresponding return period) of annual maximum temperatures over durations between one to ten days. We use multivariate copula functions to link heat wave durations and intensities. The use of these functions allows for presentation of heat wave frequency information with different combinations of intensity and severity. Copulas have been used for linking different features of drought and precipitation extremes such as duration and severity^36–40^. In this paper, we consider heat wave intensity as the average of mean daily temperature throughout the duration of heat wave. We use mean temperatures (instead of maximum temperature) is to account for night-time cooling (or lack of it), since the cumulative impacts of high temperatures (e.g. the lack of night-time cooling during a heat wave) can be detrimental to human health. Our results portray and compare HIDF curves for six cities in the United States, using daily mean temperature data from 1979–2016. We also compare HIDF curves generated from historical model simulations (including anthropogenic emissions) against natural-only historical (e.g., pre-industrial emissions level) model simulations to investigate the impacts of anthropogenic emissions on extreme heat events, using daily mean temperature data from 1850–2005. Using this approach, we concurrently compare the differences in heat wave intensity, duration, and frequency for a more comprehensive analysis of anthropogenic climate change impacts on heat waves.

## Results

**Figure 1:**
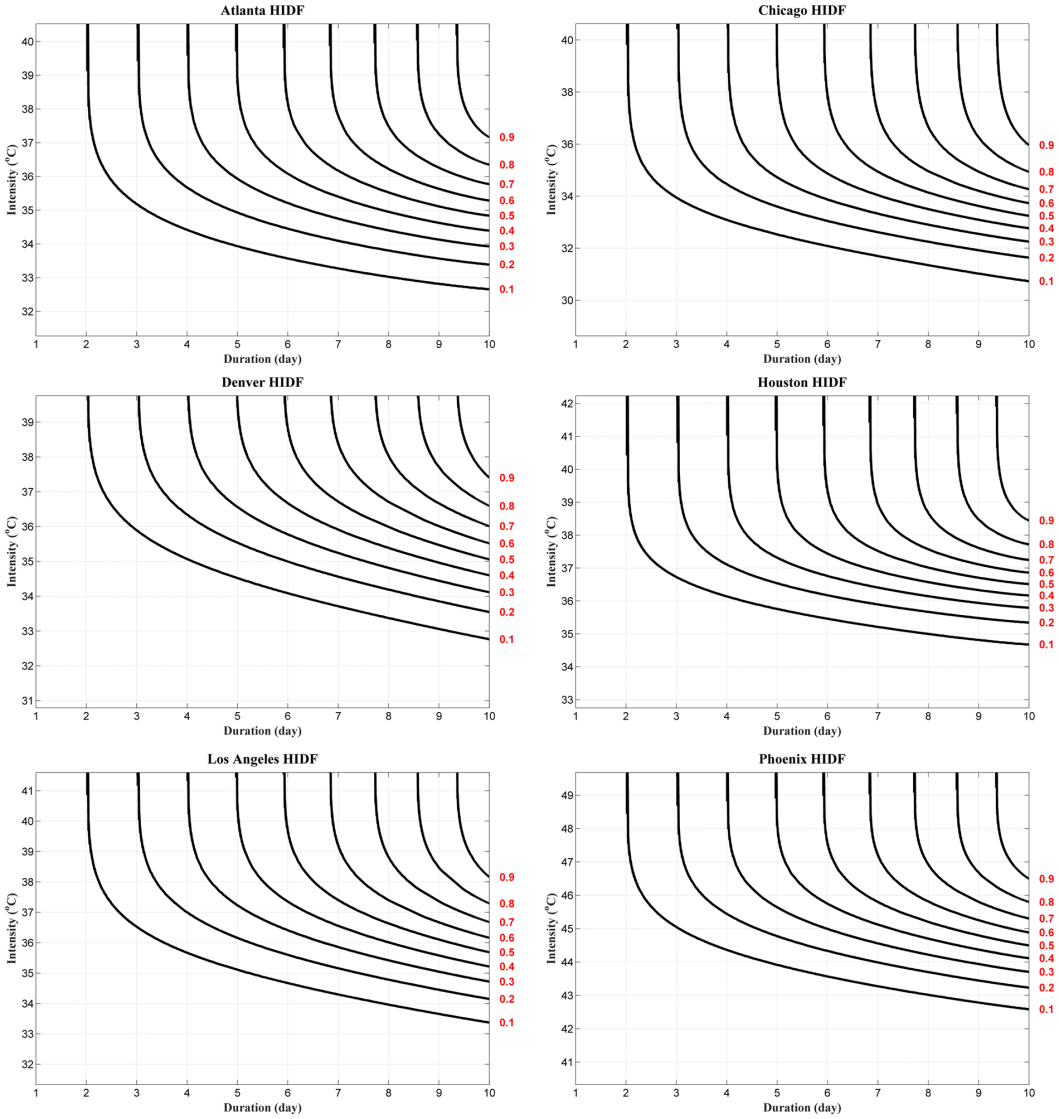
Heat wave intensity-duration-frequency (HIDF) curves for six major cities across the United States (Atlanta, Chicago, Denver, Houston, Los Angeles, and Phoenix) using non-exceedance probabilities. The red values on the right axis represent non-exceedance probabilities.

**Figure 2:**
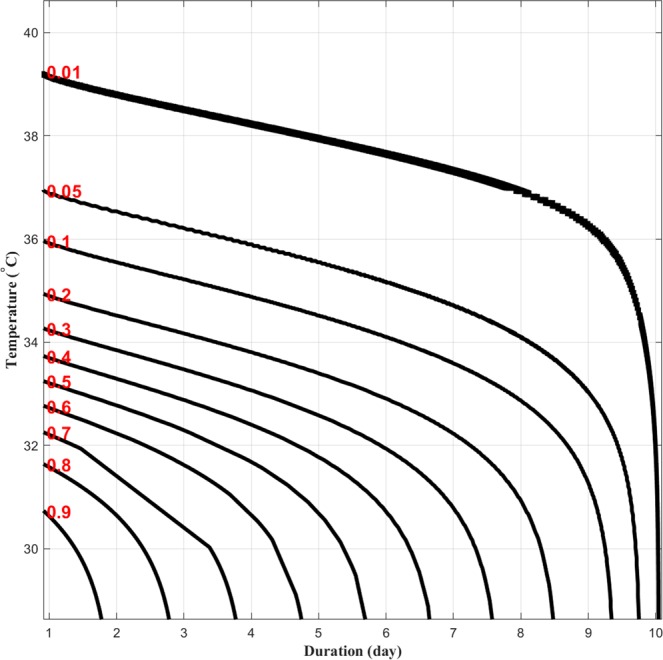
Heat wave intensity-duration-frequency (HIDF) curves for Chicago using exceedance probabilities. The red values on the left axis represent exceedance probabilities.

Figure 1 shows the heat wave IDF curves for Atlanta, Chicago, Denver, Houston, Los Angeles, and Phoenix. Each subplot depicts the joint non-exceedance probabilities for different combinations of heat wave duration and intensity. In addition to providing heat wave hazard (frequency) information, HIDF curves can be used for comparing the hazard of heat waves in different locations. For example, Fig. 1 portrays that Chicago heat waves with durations of up to six-days and average temperatures of 38 °C or less correspond to a 2-year return period (probability = 0.5), while a similar six-day heat wave with a 2-year return period in Phoenix would have a much higher intensity of 47.5 °C (see the OR hazard scenario entailed by Eq. 3, and the related references concerning the calculation of multivariate return periods). Similar comparisons can be made based on the duration or frequency of events in different regions. Figure 1 demonstrates the flexibility of the proposed HIDF curves for describing the probability of occurrence of different combinations of heat wave duration and intensity. The figure also shows different combinations of heat wave duration and intensity that lead to the same return period. For example, Fig. 1 shows that a 7-day heat wave with an intensity of 47 °C is equally likely as a 10-day heat wave with an intensity of 45 °C (here, both are 2.5 year events) in Phoenix, AZ. As mentioned in the Methods Section, the HIDF can be described based on the concept of joint exceedance probabilities (instead of non-exceedance probabilities) for different combinations of heat wave intensity and duration (Eq. 4). Figure 2 shows an example of HIDF based on the joint exceedance probabilities for the city of Chicago. The figure shows that joint exceedance probabilities of a heat wave event lasting six days or more with an average temperature exceeding 38 °C in Chicago has a return period greater than 100 years (less than 0.01 exceedance probability).

As mentioned earlier, HIDF curves can be derived based on joint exceedance probability (AND hazard scenario; see Eq. 4). Figure 2 presents a HIDF analysis based on joint exceedance probabilities (i.e., both intensity and duration being above their thresholds). HIDF curves displaying exceedance probabilities for different cities as well as CMIP5 historical and natural-only model simulations are presented in Supplementary Materials (Figs S12–S21).

**Figure 3:**
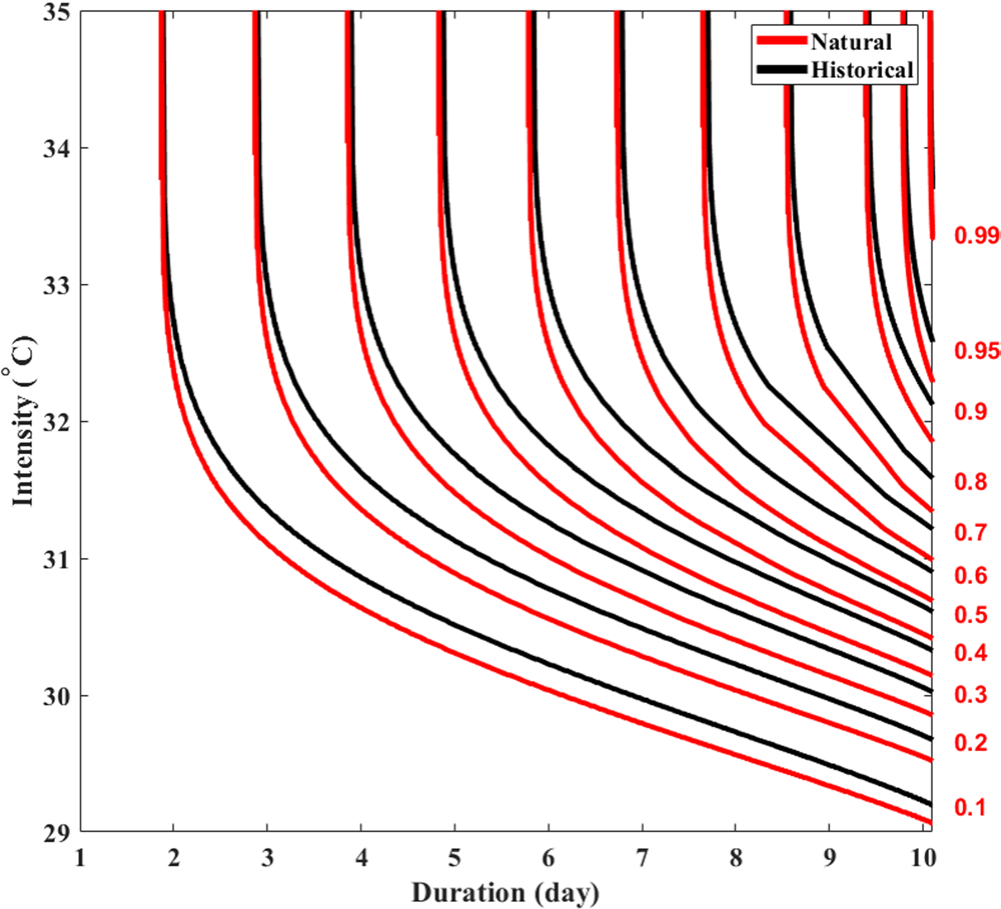
Mean heat wave intensity-duration-frequency (HIDF) curves for historical (including anthropogenic forcing) vs natural-only historical simulations from selected CMIP5 models using non-exceedance probabilities in Los Angeles, CA. The red values on the right axis represent non-exceedance probabilities.

In addition to describing characteristics of heat waves in different locations, the HIDF can be used to investigate how heat wave features have changed over time, or in response to other factors (e.g., anthropogenic emissions). Here we employ HIDF curves to attribute changes in heat wave intensity, duration, and frequency to anthropogenic emissions. Figure 3 compares HIDF curves in Los Angeles, CA using the mean of the historical (black) and natural-only (red) CMIP5 simulations. Figure 3 shows differences in the joint non-exceedance probability of heat wave duration and intensity between historical and natural-only simulations (1950–2005). This figure demonstrates that heat wave events are generally shorter and less intense under the natural-only forcing (without anthropogenic emissions) in relation to the historical forcing (with anthropogenic emissions). In other words, a heat wave with the same intensity and duration under natural-only historical conditions has a lower frequency (and probability of occurrence) than a heat wave occurring under historical conditions perturbed by anthropogenic emissions. For example, this figure shows that an extreme ten-year, ten-day heat wave event have an intensity of 31.9 °C under natural-only conditions, while the event would have an intensity of 32.2 °C under historical conditions. This is a statistically significant difference at 0.05 significance level (see Supplementary Materials). Mazdiyasni *et al*., 2017 show that an increase of only 0.5 °C in summer mean temperatures leads to a 146% increase in the probability of mass mortality events in India. The study also shows that an increase of heat wave days from six to eight days over the summer season leads to an 84% increase in the probability of mass mortality events. We can infer than the effects of a rise of 0.3 °C in a 10-year heat wave event over 10 consecutive days will have significant implications on human health, agriculture, the environment, and the electric grid^7^. Figure S1, in Supplementary Materials, portrays the HIDF curves of the individual climate models.

**Figure 4:**
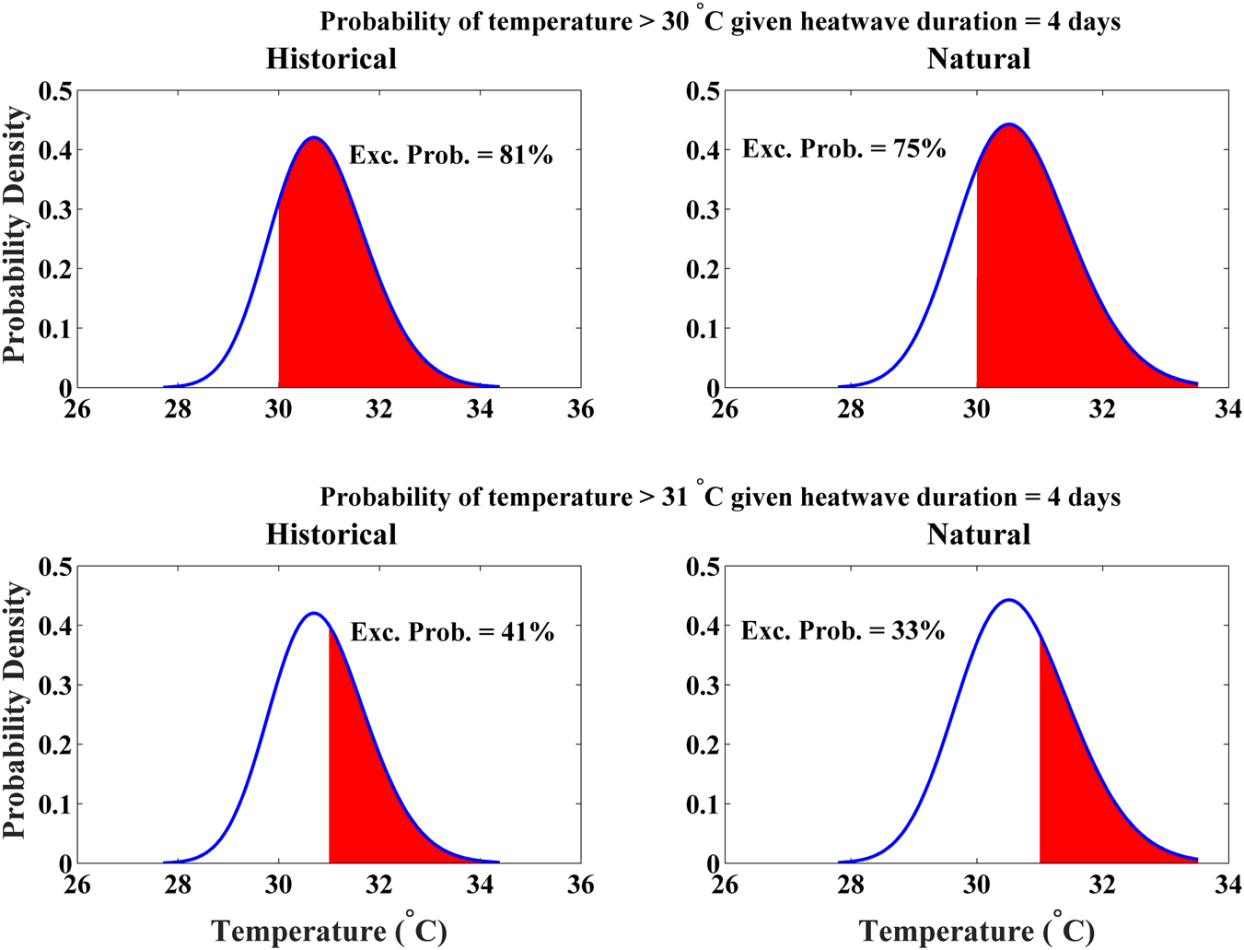
Comparison between historical (including anthropogenic forcing) vs natural-only historical parametric conditional probability density functions (PDFs) using the mean of CMIP5 simulations for heat wave intensity given heat wave duration equal to four days in Los Angeles, CA.

Given that the HIDF curve is based on a multivariate framework, we can extract information about one variable (e.g., heat wave intensity) conditioned on a second variable (e.g., heat wave duration) – Eq. 5. Figure 4, for example, displays the difference in heat wave intensity given heat wave duration between historical vs natural-only conditions, using the mean of the four CMIP5 models (Fig. S2 shows similar results for each individual CMIP5 model). We show that the probability of heat wave intensity being greater than 30 °C given a duration of four days is eight percent greater under historical conditions in comparison to natural-only conditions (81% vs 75%). We also show that the probability of intensity being greater than 31 °C given a heat wave duration of four days is 24 percent greater under historical conditions vs natural-only conditions (41% vs 33%, respectively). Note that we are considering relative percent change with respect to natural-only conditions. These increases in the probability of intense heat over several consecutive days can be attributed to anthropogenic warming. Figure S3 also shows similar results for six-day heat waves. As shown, anthropogenic warming has increased the likelihood of a six-day heat wave (temperature > 30 °C) by 10% and a six-day heat wave (temperature > 31 °C) by 29%. Figures 4 and S3 imply greater increases in the likelihood of longer and more intense heat waves in the historical simulations relative to the natural-only. Therefore, we can conclude that greater increases in the likelihood of extreme (in intensity and duration) heat wave events may have been driven by anthropogenic warming.

## Discussion

Global warming is causing an increase in the frequency and severity of heat wave events, which increases the importance of using a robust model in understanding heat waves and quantifying heat wave properties. We propose a multivariate approach to construct heat wave intensity, duration, frequency (HIDF) curves, which enables robust frequency (hazard) analysis of extreme heat events, while concurrently accounting for both intensity and duration of heat waves. Since these heat wave features are interdependent, it is important to model the relationship in a manner that avoids biases in the frequency analyses. The main objective of this paper is to present the HIDF methodology and show different types of applications including describing heat wave features and conducting attribution analyses. An attribution analysis using the proposed HIDF curves shows that the anthropogenic emissions have increased the likelihood of a four-day heat wave (temperature > 31 °C) by 24%. We further show a six-day heat wave (temperature > 31 °C) has a 29% higher likelihood under the anthropogenic emission scenario relative to the natural-only scenario. The proposed method is general and can be applied to different locations and different combinations for heat wave durations and intensities.

The HIDF curve can potentially be used by for design of infrastructure systems such as electric grids and power plants. For example, an electric grid is designed for peak demand and must consider both the duration and intensity of heat wave events simultaneously, similar to the use of precipitation IDF curves for highway culvert design. Although analyzing heat waves with univariate indices have provided useful information in the past, our proposed metric can change how we view the extremeness of a heat wave event moving forward.

## Methods

We create the HIDF curves by determining the non-exceedance probability of heat wave duration and intensity, although HIDF can also be defined based on exceedance probability. We define heat wave intensity using the average daily temperature throughout the duration of the event. The HIDF model differs from the typical heat wave definition as it lacks a certain temperature threshold to define heat waves. Instead, similar to traditional precipitation IDF curves, we use the block maxima method to produce the HIDF curves. We determine the hottest heat wave events in each year with durations ranging from one to ten consecutive days. We do this by first determining the hottest, two consecutive hottest, three consecutive hottest, …, ten consecutive hottest days for each year, and then modeling the joint probabilities of all those block maxima temperature values with their corresponding durations. We consider the highest mean temperature over the duration period. We subsequently calculate the intensity of each event using,

1 $$\forall y\,{I}_{y}=\,\max \{\frac{{\sum }_{i}^{i+D-1}{t}_{i}^{y}}{D}\},$$

$$\forall D\,D=1:10,$$

where *y* is the year, *i* is the first day in the moving window that ranges between one and the number of days in the year, ${t}_{i}^{y}$ is the average daily temperature at day *i* of year *y*, and *D* is the length of the running window (i.e. heat wave duration). Note that the data used to create HIDF curves consists of heat waves with one, two, …, ten days duration and associated average daily temperature during the event. Hence, each year in the period of observation yields 10 pairs of duration and intensity data.

In other words, we first determine the average temperature of the one to ten hottest consecutive days in each year to model HIDF curves. We then use multivariate copula functions to find the non-exceedance (or exceedance) joint probability cumulative distribution function of heat wave duration and intensity^41–46^. We first determine the admissible models via suitable Goodness-of-Fit tests, at 0.05 significance level, out of the 25 copula families and the 17 distributions built into the Multivariate Copula Analysis Toolbox. Then, we choose the best fitting (admissible) model based on the Bayesian Information Criterion^47,48^, based on the Bayesian Information Criterion which could not be rejected as determined by the associated p-values at 0.05 significance level^49–51^. Refer to Table S1 of^47^ for more information regarding the copula families and find the list of marginal distributions used in this study in the Supplementary Information (SI). Also see the Supplementary Table S2 for information on the metrics of the best fitting copula and marginal distributions. Table S2 shows a summary of the selected marginal distributions, best-fitted copula families, and their performance in terms of Root Mean Square Error (RMSE) and Nash-Sutcliffe Efficiency (NSE). We acknowledge that duration cannot be considered as a pure random variable which may affect the results. Given that the marginal distribution for duration is a non-continuous function, we added a small random noise; i.e. Gaussian distributed perturbations centered on the data with a standard deviation of 0.05^52–54^. This makes the marginal cumulative distribution a continuous function and randomizes the duration variable. Figures S4–S11 show that there is no significant change in the multivariate cumulative distribution function when adding noise to heat wave duration (continuous function) as compared to the original discrete distribution. Another method to transform the marginal cumulative distribution into a continuous function is to add uniform perturbations centered on the data along (−0.5, 0.5)^52,55,56^. We have also performed this analysis, and included it in Figs S29–S38.

We can specify a probability model for dependent multivariate observations, by expressing the *d* dimensional joint cumulative distribution *F* in terms of its marginals *F*_1_,*…F*_*d*_, and the associated copula ***C***, following Sklar’s theorem^57,58^.

2 $${\boldsymbol{F}}({x}_{1},\ldots ,{x}_{d})={\boldsymbol{C}}({F}_{1}({x}_{1}),\ldots ,{F}_{d}({x}_{d}))$$

We use bivariate copula to estimate HIDF curve by calculating the joint non-exceedance probability distribution of heat wave duration ($X$), and intensity ($Y$),

3 $$P({X}_{1} > {x}_{1}{\cup }^{}{X}_{2} > {x}_{2})=1-{\boldsymbol{F}}({x}_{1},\,{x}_{2})=1-{\bf{C}}[{{\rm{F}}}_{1}({x}_{1}),\,{{\rm{F}}}_{2}({{\rm{x}}}_{2})]$$

We then calculate the joint return periods for different duration and frequency following^48^, first outlined in^59^, and mathematically formalized in^60^. This formulation is also known as the OR hazard scenarios in which either duration or intensity exceed their corresponding thresholds.

We can also derive HIDF curves by calculating the joint exceedance probabilities (also known as AND hazard scenario), as^61^

4 $$\begin{array}{c}P({X}_{1} > {x}_{1}{\cap }^{}{X}_{2} > {x}_{2})\\ \,=1-{{\rm{F}}}_{1}({{\rm{x}}}_{1})-{{\rm{F}}}_{2}({{\rm{x}}}_{2})+{\boldsymbol{F}}({x}_{1},\,{x}_{2})\\ \,=1-{{\rm{F}}}_{1}({{\rm{x}}}_{1})-{{\rm{F}}}_{2}({{\rm{x}}}_{2})+{\bf{C}}[{{\rm{F}}}_{1}({{\rm{x}}}_{1}),{{\rm{F}}}_{2}({{\rm{x}}}_{2})].\end{array}$$

Both HIDF derivations (exceedance vs non-exceedance joint probabilities) are acceptable for determining joint return periods. We show example applications based on both approaches, however, we estimate HIDF using joint non-exceedance probabilities in the majority of the analyses conducted in this paper.

We also determine the conditional density function of heat wave intensity at a certain duration (X_1_ = x_1_), that is ${{\rm{f}}}_{{{\rm{X}}}_{2}|{{\rm{X}}}_{1}}({{\rm{x}}}_{2}|{{\rm{x}}}_{1})$ through^62,63^:

5 $${{\rm{f}}}_{{{\rm{X}}}_{2}|{{\rm{X}}}_{1}}({{\rm{x}}}_{2}|{{\rm{x}}}_{1})={\bf{c}}[{{\rm{F}}}_{1}({{\rm{x}}}_{1}),\,{{\rm{F}}}_{2}({{\rm{x}}}_{2})].{{\rm{f}}}_{{{\rm{x}}}_{2}}({{\rm{x}}}_{2})$$

using non-exceedance joint probabilities, in which, **c** is the copula probability density function (PDF) and ${{\rm{f}}}_{{{\rm{X}}}_{2}}({{\rm{x}}}_{2})$ is the heat wave intensity density function. Once we construct a conditional marginal PDF from Eq. 5, we can calculate the probability of intensity (X_2_) exceeding a particular threshold (x_2_) from the area under the curve, delineated by: ${f}_{{X}_{2}|{X}_{1}}({X}_{2} > {x}_{2}\,|\,{x}_{1})$. We apply this technique to calculate ${f}_{{X}_{2}|{X}_{1}}({x}_{2}\,|\,{x}_{1})$ for different values of x_1_ (e.g., duration = 5 days).

## Data

The proposed methodology is generalized and can be applied to different locations. Here, we use daily average temperatures for Atlanta (33.7490°N, 84.3880°W), Chicago (41.8781°N, 87.6298°W), Denver (39.7392°N, 104.9903°W), Houston (29.7604°N, 95.3698°W), Los Angeles (34.0522°N, 118.2437°W), and Phoenix (33.4484°N, 112.0740°W) from the Climate Prediction Center (CPC) global air temperature dataset provided by NOAA/OAR/ESRL PSD, Boulder, Colorado, USA (http://www.esrl.noaa.gov/psd/). This dataset includes near surface air temperature with a 0.5 degree spatial resolution and daily temporal resolution. We interpret observed temperature data from the grid encompassing each city to be representative of that particular city.

For attribution analysis, we use the Coupled Model Intercomparison Project Phase 5 (CMIP5) historical and natural-only historical simulations from 1850–2005 to quantify the impact of anthropogenic climate change on daily average temperature values^64^. CMIP5 is an ensemble of climate model experiments intended to improve our understanding of pre-industrial, historical, and projected climate^64^; the spatial resolution of the models used in our study are listed in Table S1. The historical experiment imposes conditions – such as anthropogenic and natural trends and variability – that reflect what has been seen in the observations, including changes in atmosphere due to human and volcanic emissions, solar forcing, aerosols, and human land use^64^. The natural-only historical simulations capture natural trends and variability without anthropogenic forcing^64^. With the climate simulations, we could attribute differences between the two simulations to anthropogenic climate change. We show an example application for the city of Los Angeles, California (34.0522, −118.2437) using the grid cell encompassing Los Angeles from five GCMs.

## Supplementary information

Supplementary Figures

Supplementary Tables

## Data Availability

All data used in this study is open to the public.
