# Supplementary material for: Heat wave Intensity Duration Frequency Curve: A Multivariate Approach for Hazard and Attribution Analysis
Source: Sci Rep. 2019 Oct 1;9:14117. doi: 10.1038/s41598-019-50643-w (PMC6773721; doi:10.1038/s41598-019-50643-w)
Supplement: Supplementary file 1 — Supplementary Figures [file 41598_2019_50643_MOESM1_ESM.docx]

**Supplementary Information - Figures**

**Heat wave Intensity Duration Frequency Curve: A Multivariate Approach for Hazard and Attribution Analysis**

Omid Mazdiyasni^1*^, Mojtaba Sadegh^2^, Felicia Chiang^1^, Amir AghaKouchak^1, 3^

^1^ Department of Civil and Environmental Engineering, University of California, Irvine, California 92697, USA

^2^ Department of Civil and Environmental Engineering, Boise State University, Idaho 83725, USA

^3^ Department of Earth System Science, University of California, Irvine, California 92697, USA


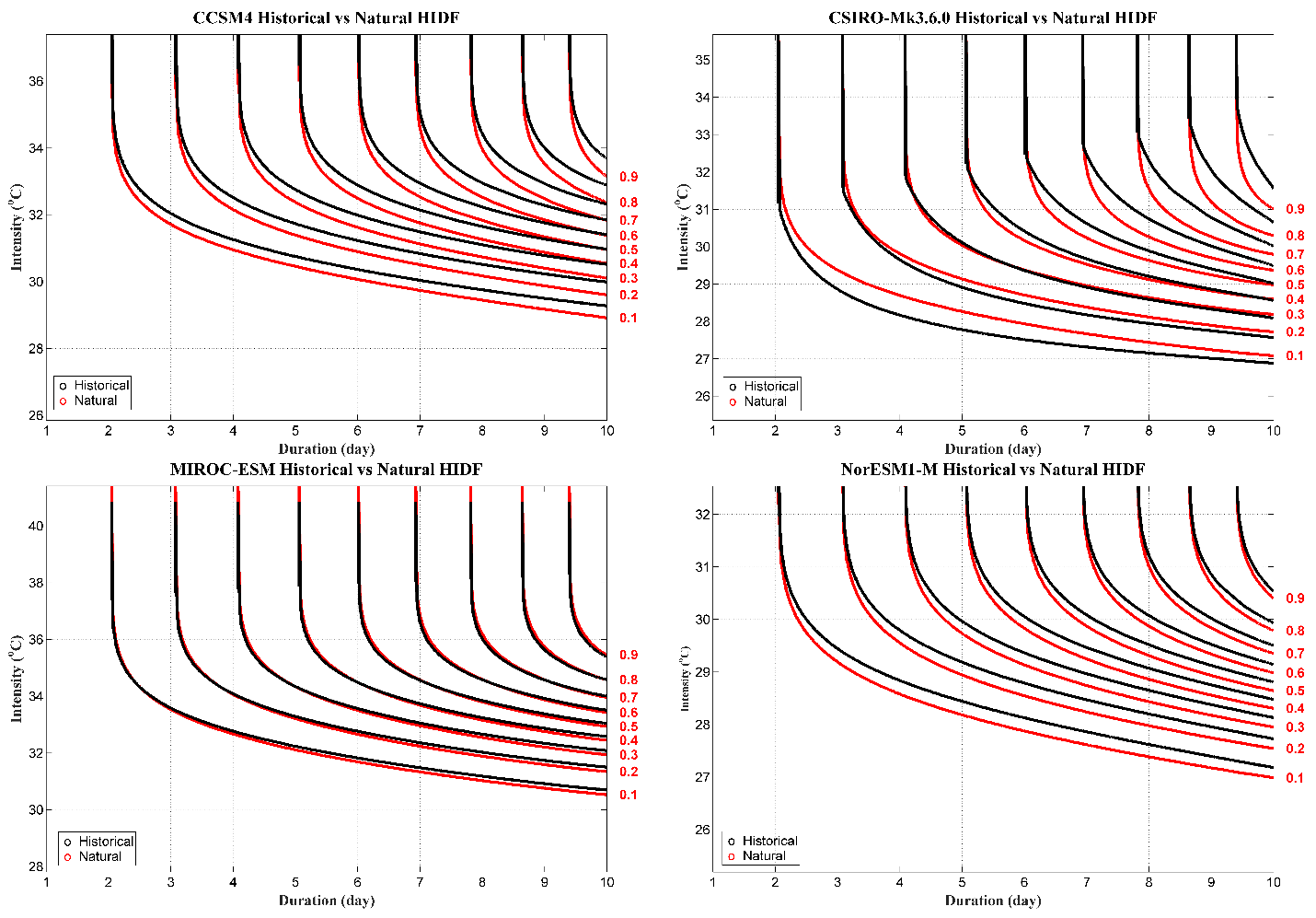


Figure S1: Heat wave intensity-duration-frequency (HIDF) curves for historical (including anthropogenic forcings) vs natural-only historical simulations from selected CMIP5 models. The red values on the right axis represent non-exceedance probabilities.


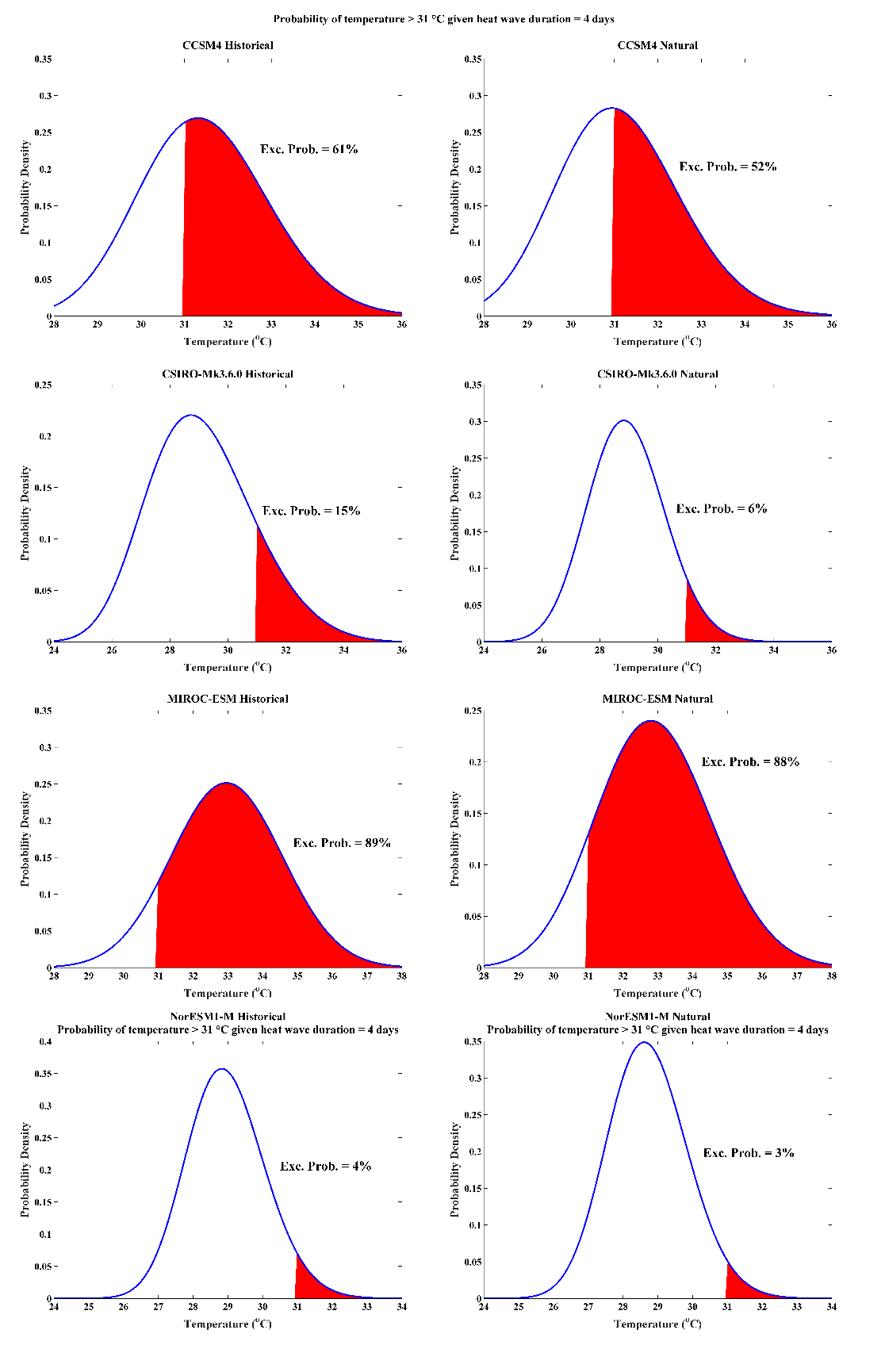


Figure S2: Comparison between historical (including anthropogenic forcings) vs natural-only historical parametric conditional probability density functions (PDFs) for heat wave intensity given heat wave duration equal to four days.


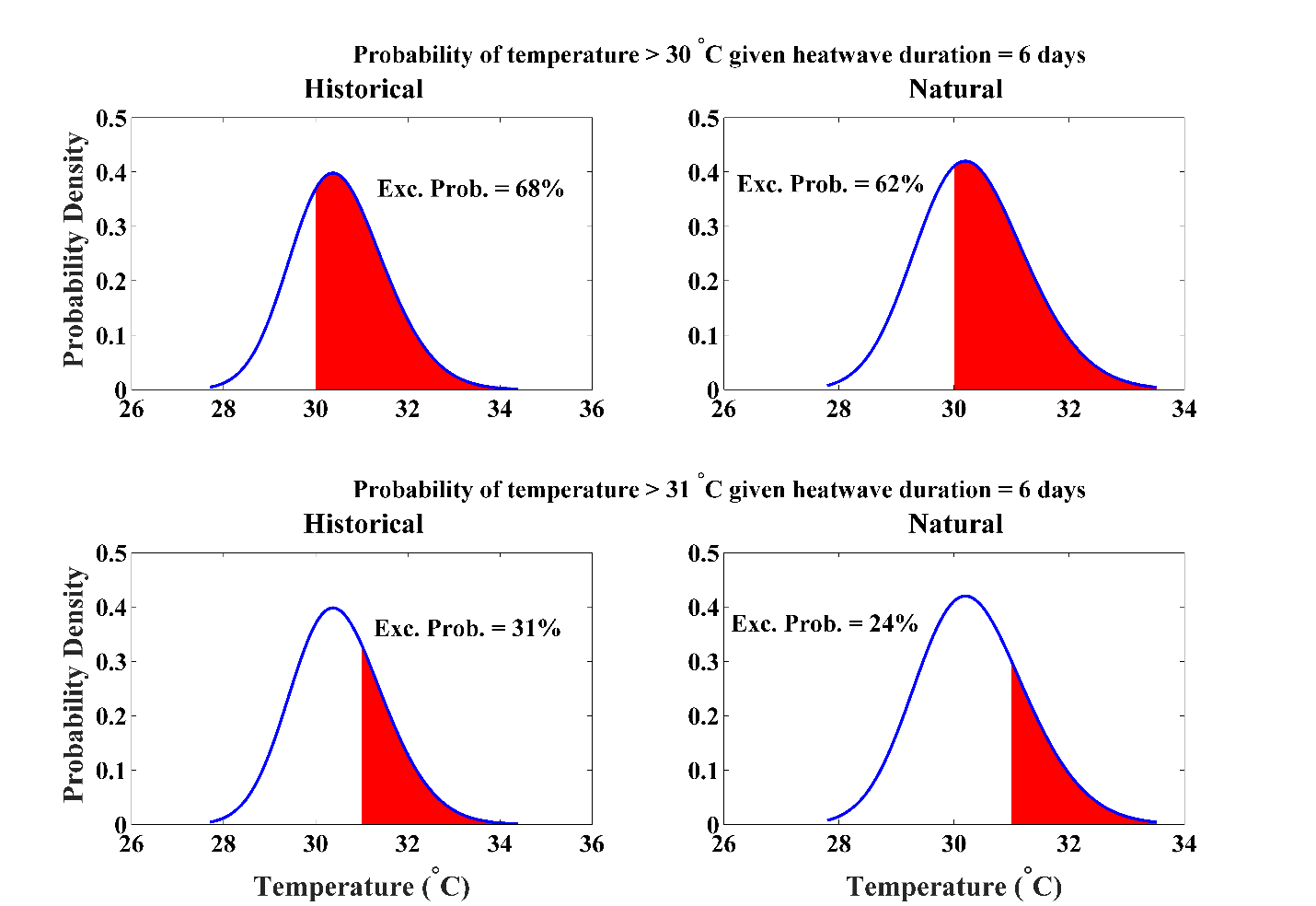


Figure S3: Comparison between historical (including anthropogenic forcings) vs natural-only historical parametric conditional probability density functions (PDFs) using the mean of CMIP5 simulations for heat wave intensity given heat wave duration equal to six days.


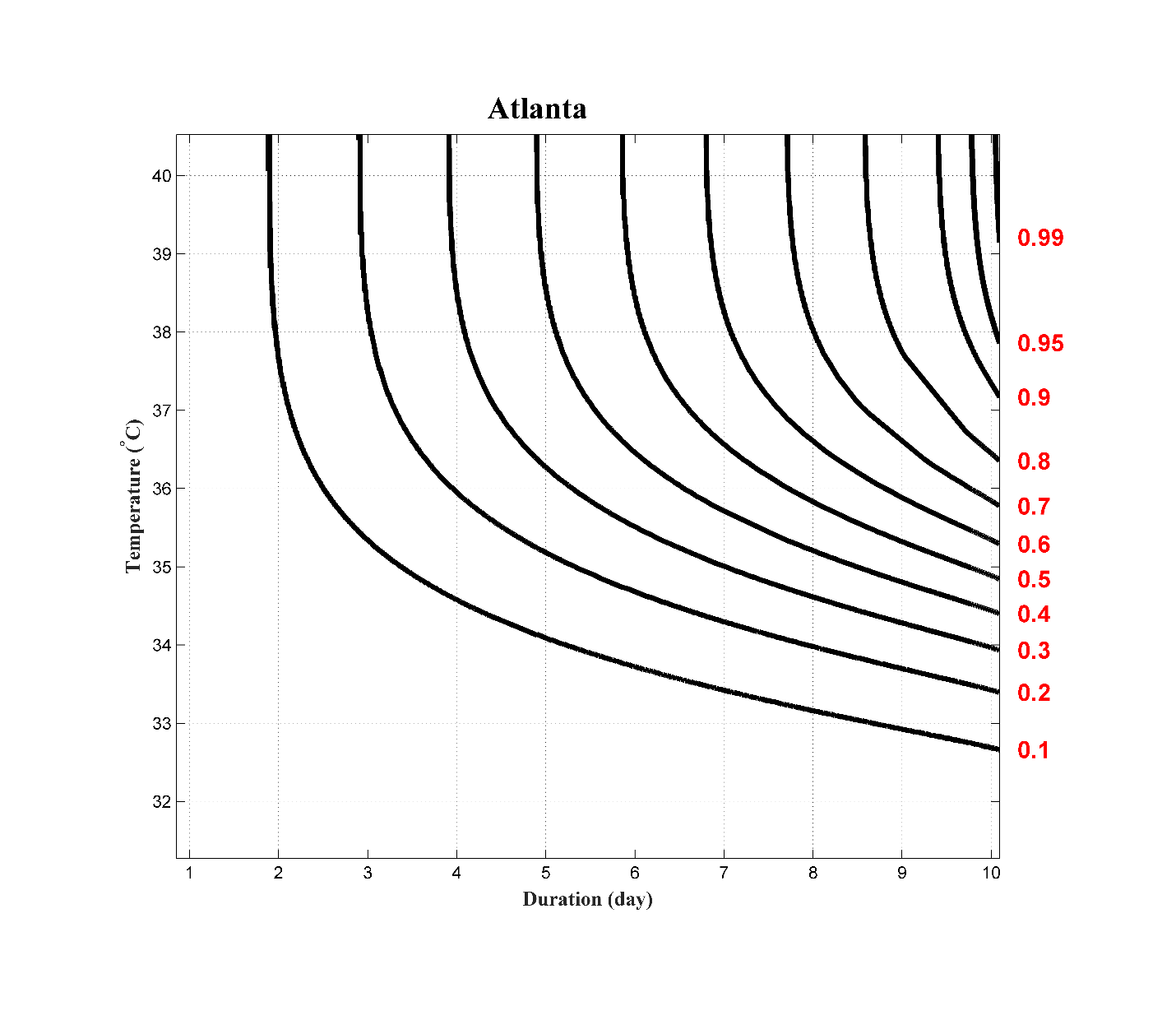


Figure S4: Heat wave intensity-duration-frequency (HIDF) curve for Atlanta including Gaussian perturbations along the duration axis, with a standard deviation of 0.05. The red values on the right axis represent non-exceedance probabilities.


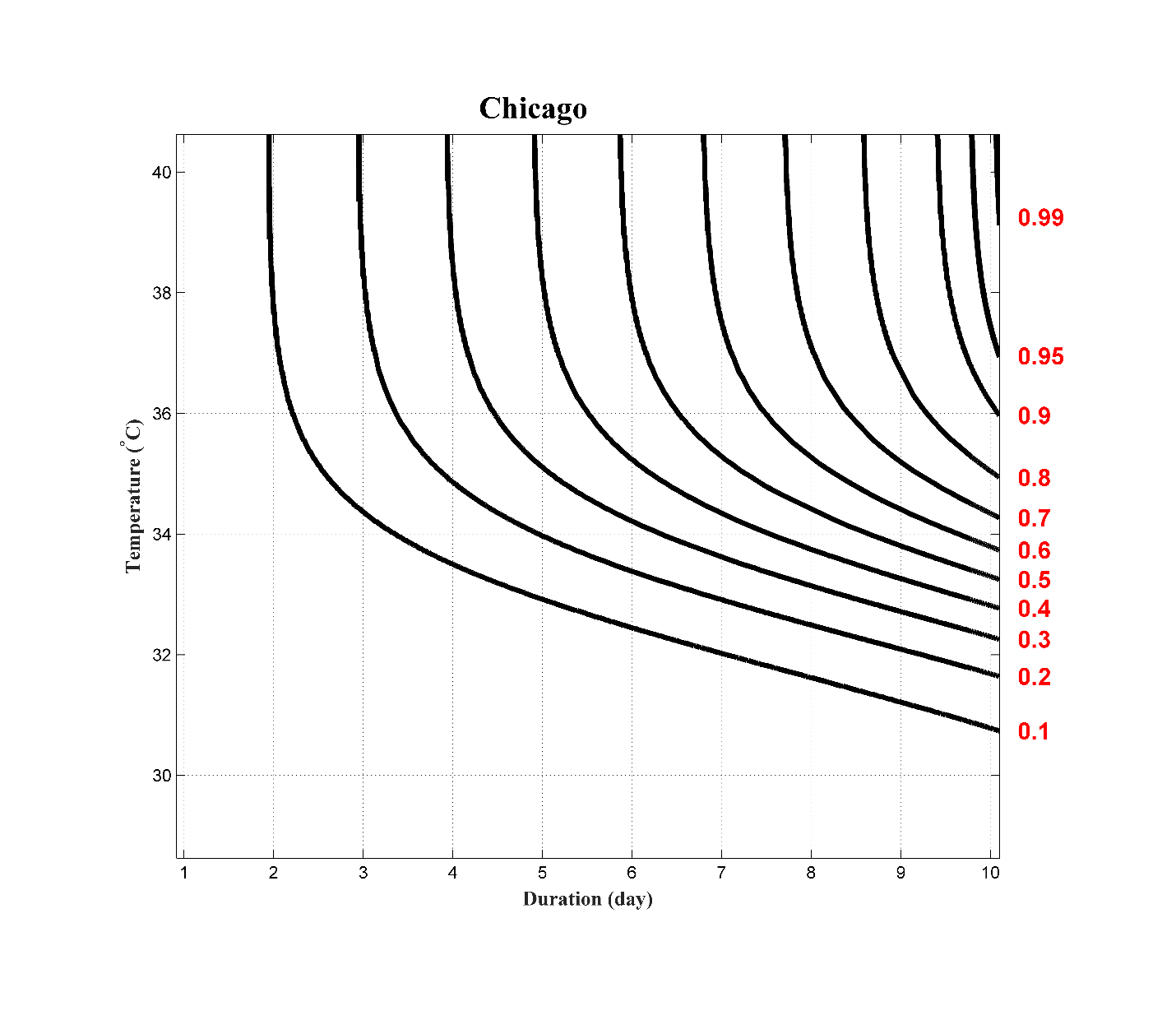


Figure S5: Heat wave intensity-duration-frequency (HIDF) curve for Chicago including Gaussian perturbations along the duration axis, with a standard deviation of 0.05. The red values on the right axis represent non-exceedance probabilities.


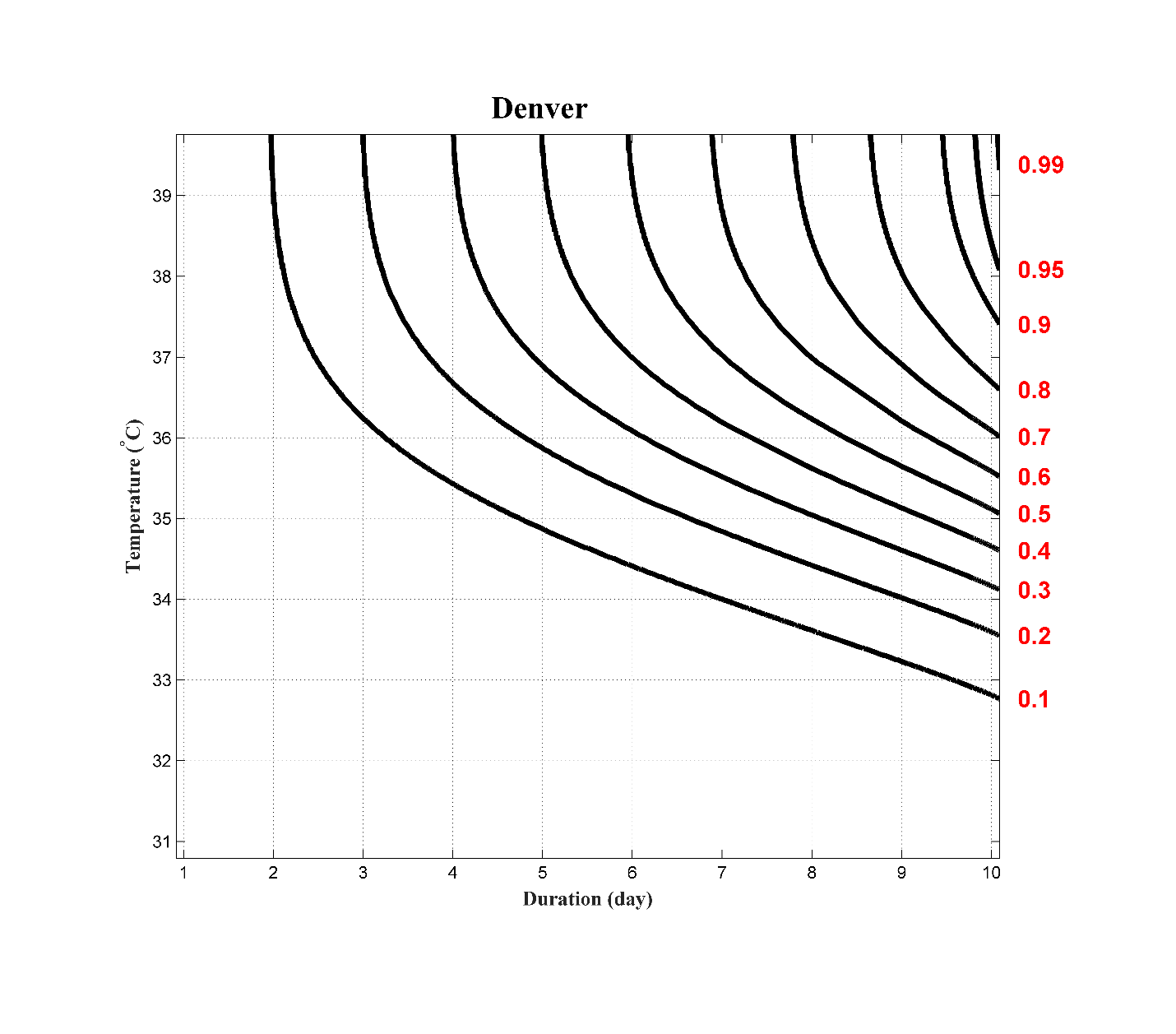


Figure S6: Heat wave intensity-duration-frequency (HIDF) curve for Denver including Gaussian perturbations along the duration axis, with a standard deviation of 0.05. The red values on the right axis represent non-exceedance probabilities.


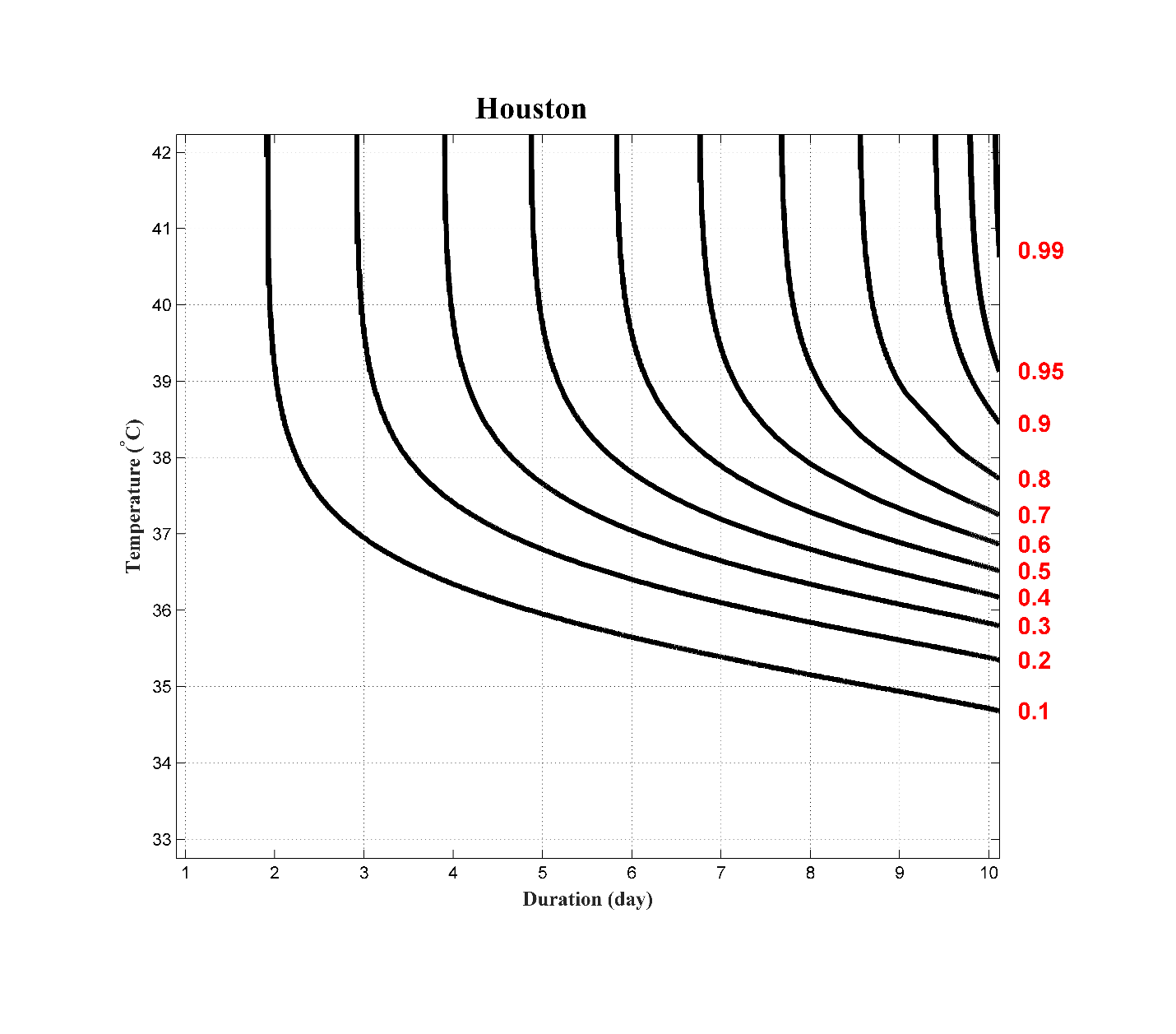


Figure S7: Heat wave intensity-duration-frequency (HIDF) curve for Houston including Gaussian perturbations along the duration axis, with a standard deviation of 0.05. The red values on the right axis represent non-exceedance probabilities.


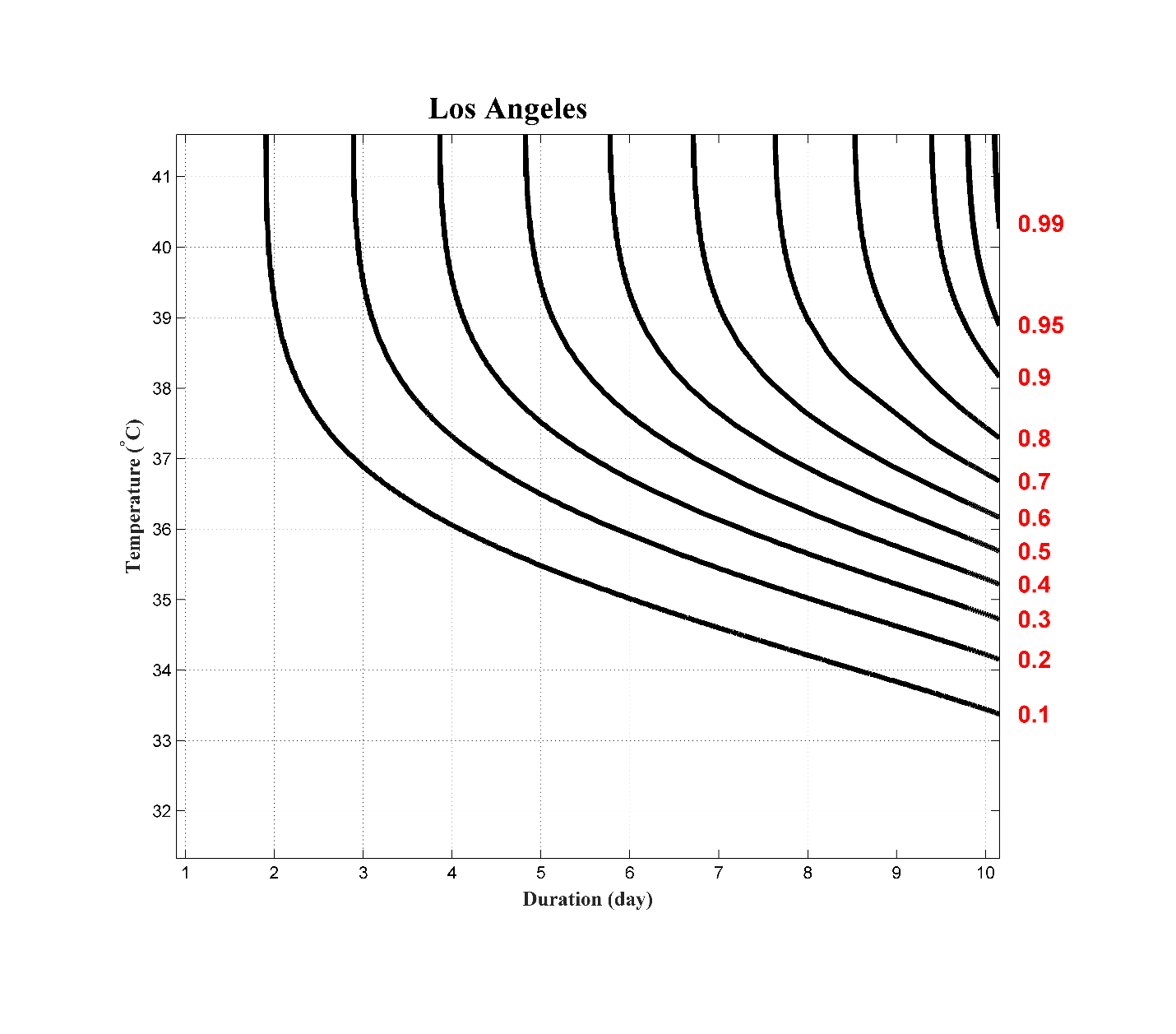


Figure S8: Heat wave intensity-duration-frequency (HIDF) curve for Los Angeles including Gaussian perturbations along the duration axis, with a standard deviation of 0.05. The red values on the right axis represent non-exceedance probabilities.


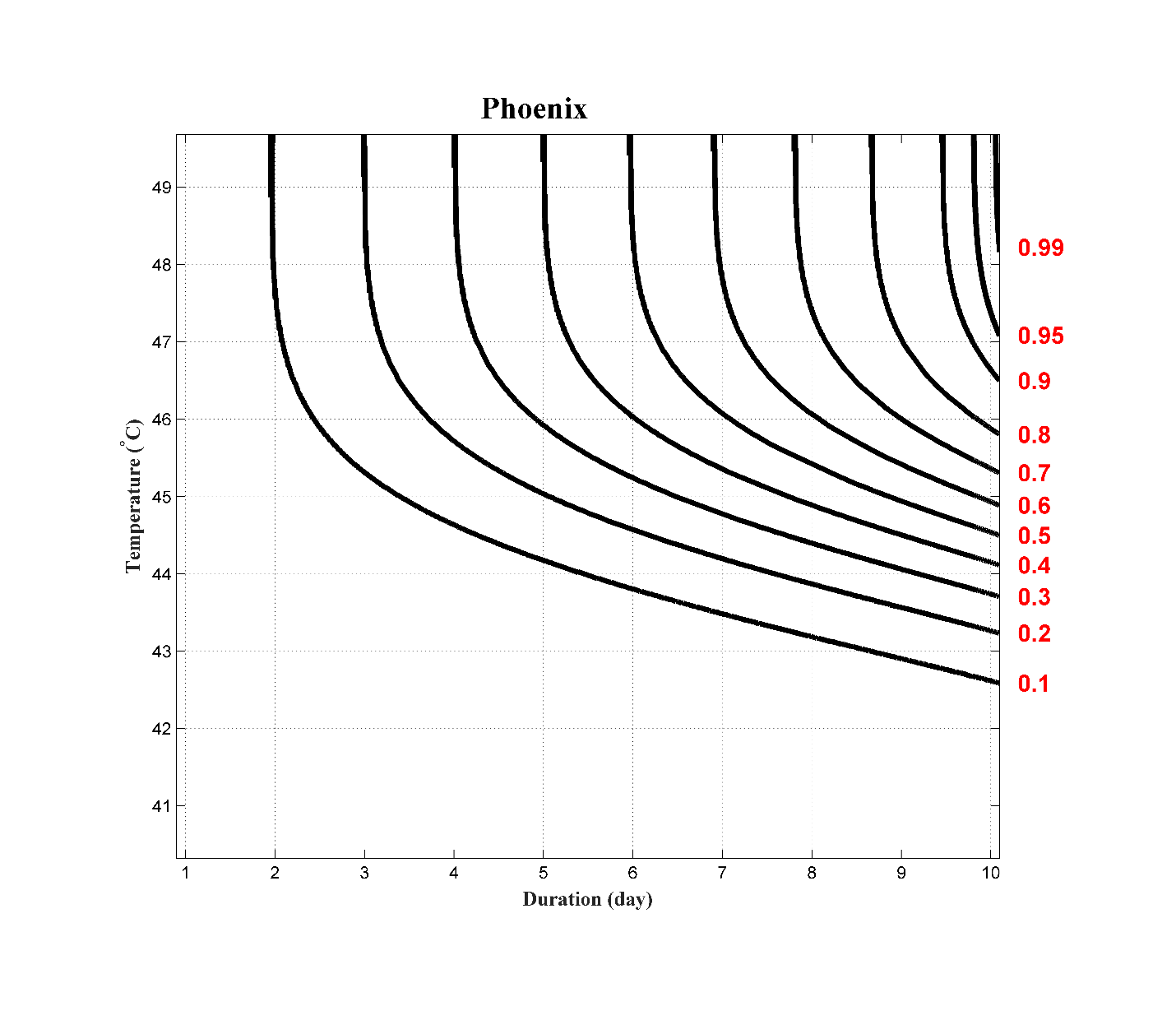


Figure S9: Heat wave intensity-duration-frequency (HIDF) curve for Phoenix including Gaussian perturbations along the duration axis, with a standard deviation of 0.05. The red values on the right axis represent non-exceedance probabilities.


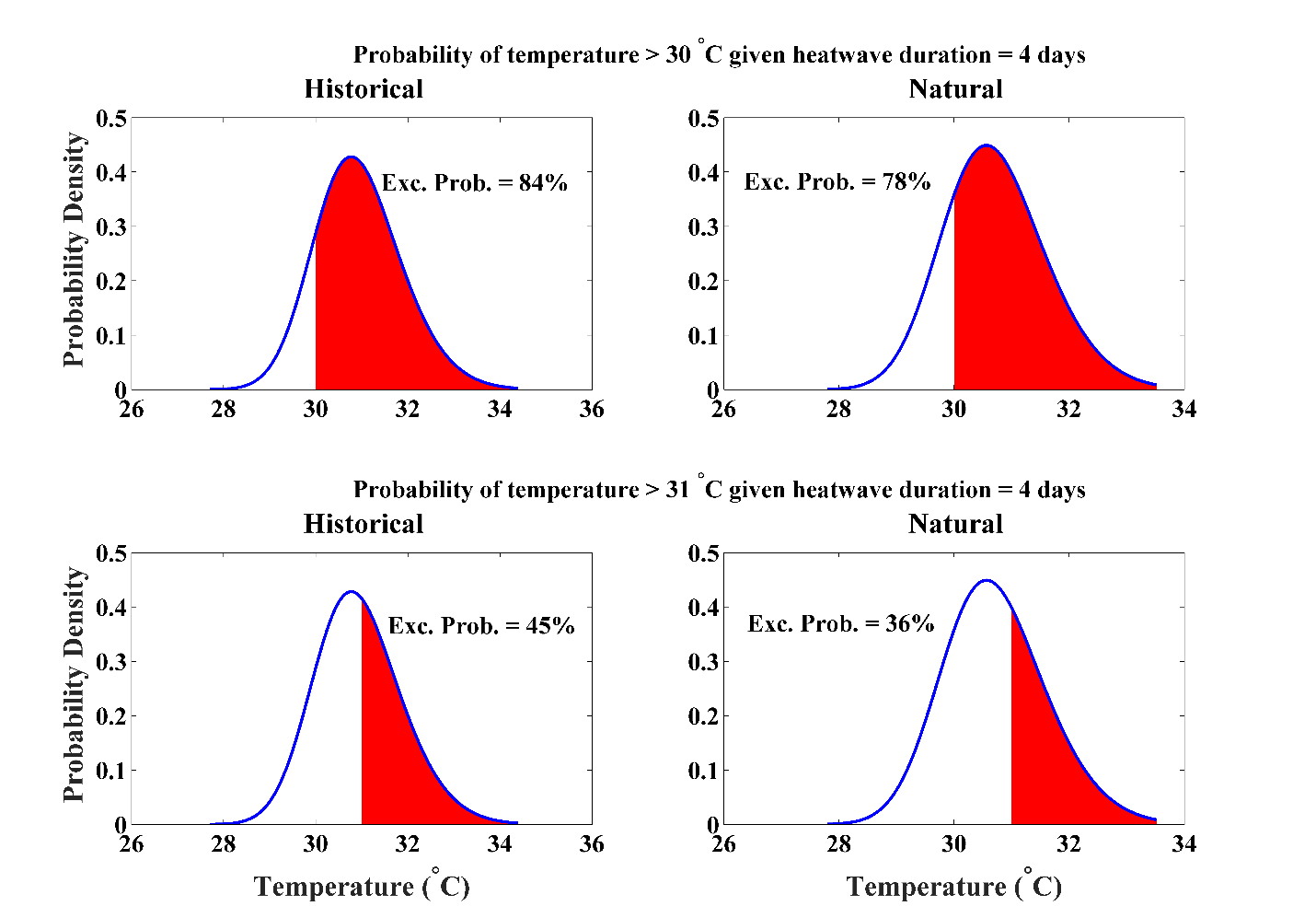


Figure S10: Comparison between historical (including anthropogenic forcings) vs natural-only historical parametric conditional probability density functions (PDFs) using the mean of CMIP5 simulations for heat wave intensity given heat wave duration equal to four days, including Gaussian perturbations along the duration axis, with a standard deviation of 0.05.


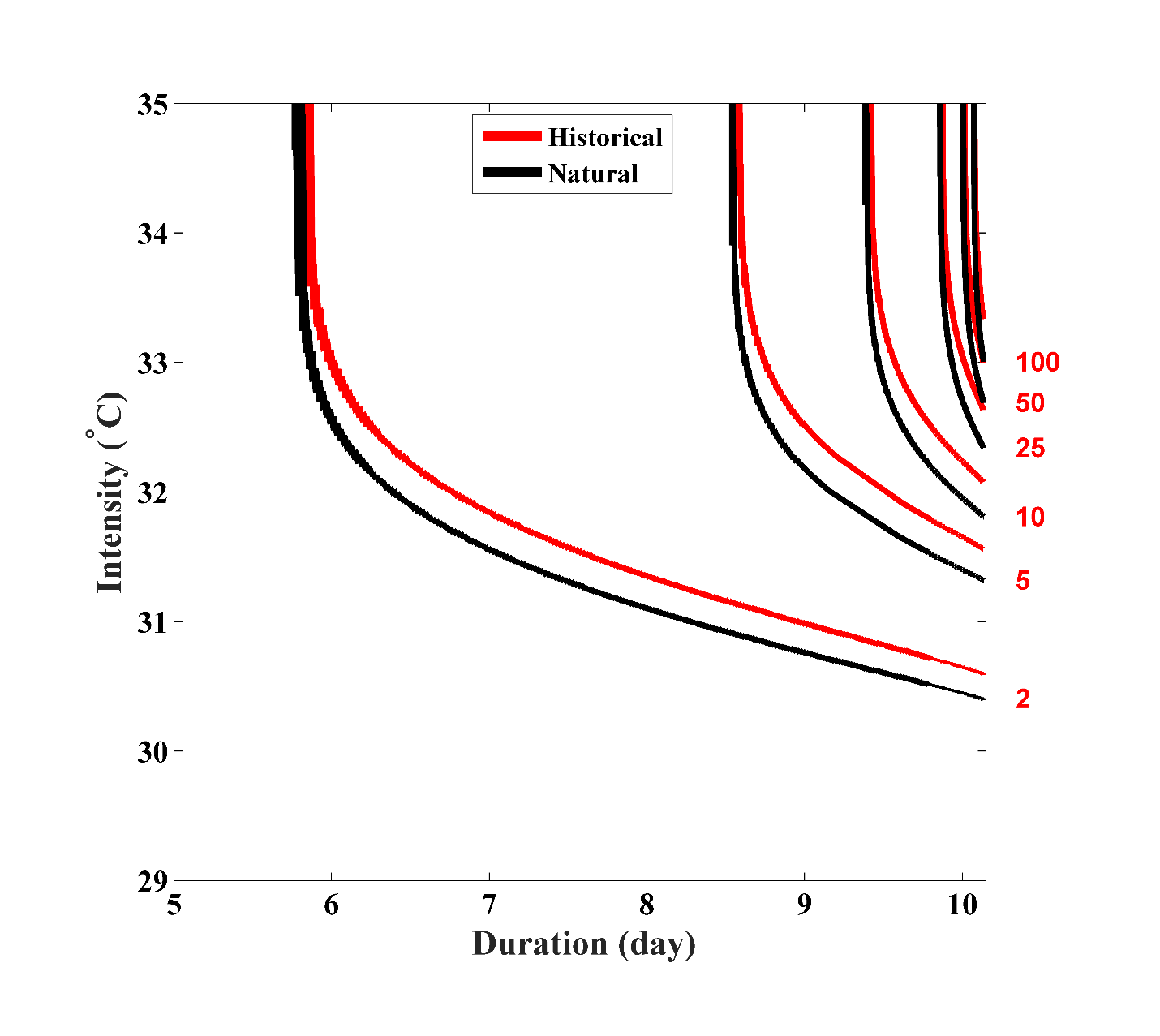


Figure S11: Mean heat wave intensity-duration-frequency (HIDF) curve for historical (including anthropogenic forcings) vs natural-only historical simulations from selected CMIP5 models including Gaussian perturbations along the duration axis, with a standard deviation of 0.05. This figure shows the frequency in terms of return period rather than non-exceedance probability. The red values on the right axis represent return periods.


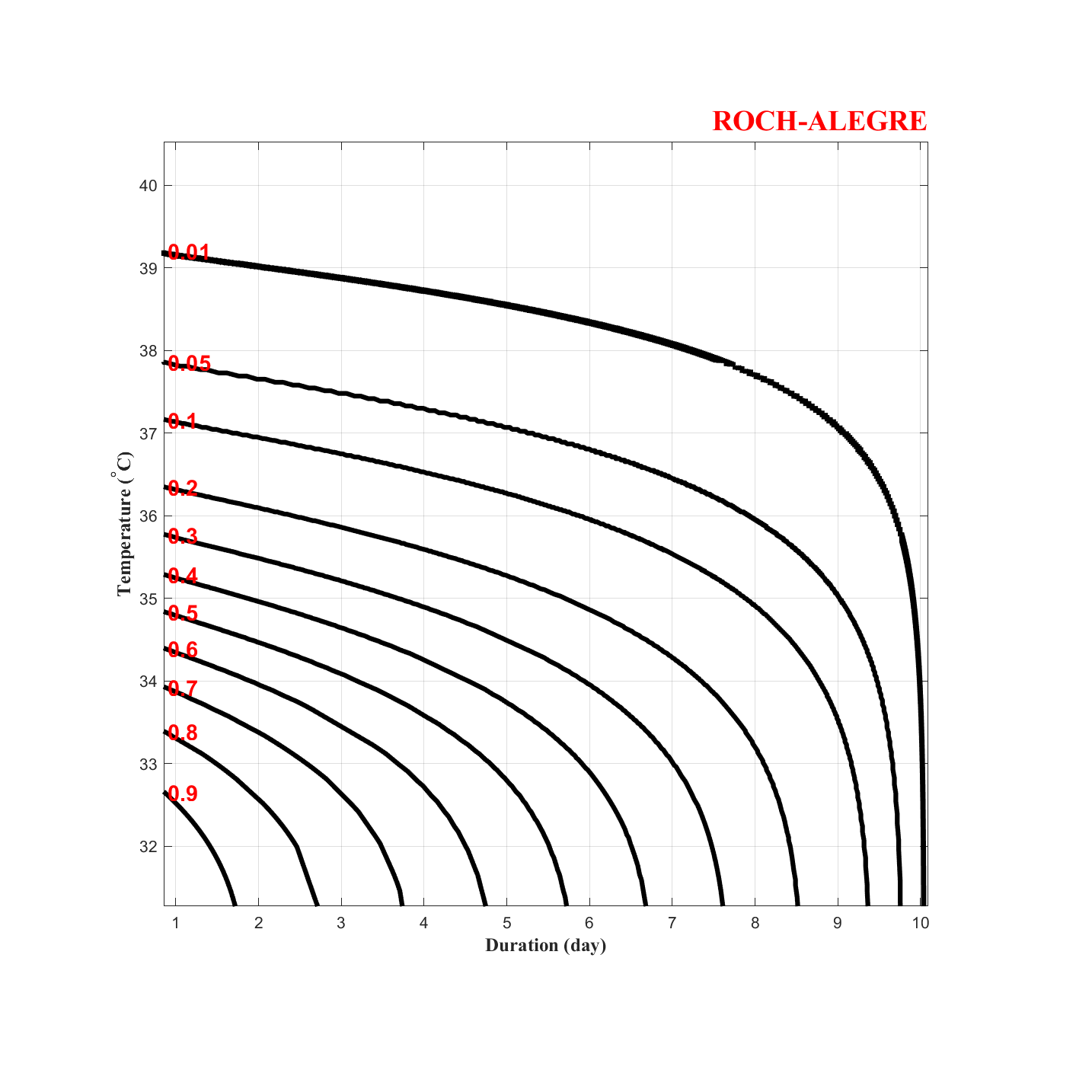


Figure S12: Heat wave intensity-duration-frequency (HIDF) curve for Atlanta including Gaussian perturbations along the duration axis, with a standard deviation of 0.05. This figure shows the frequency in terms of exceedance probability rather than non-exceedance probability. The red values on the left axis represent exceedance probabilities.


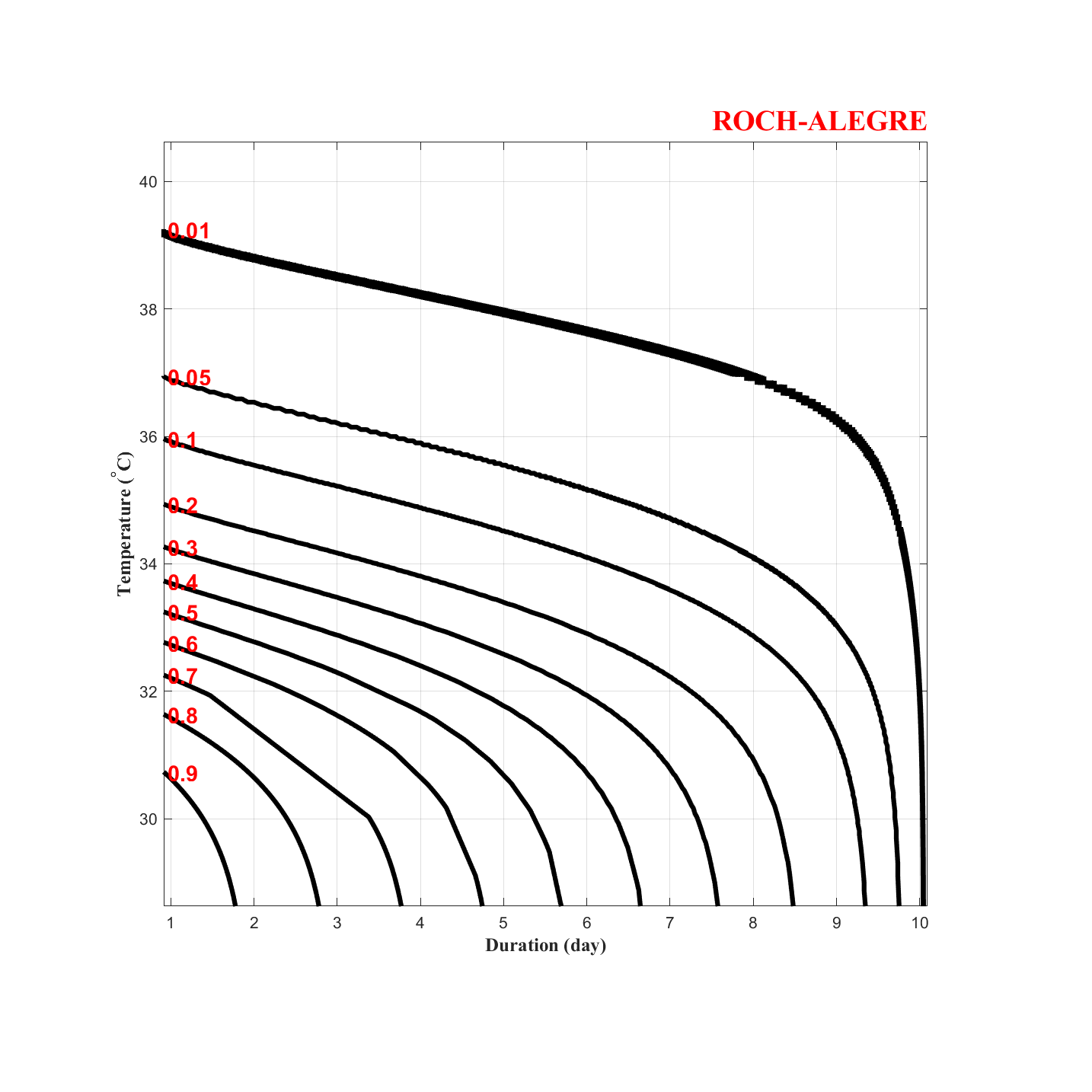


Figure S13: Heat wave intensity-duration-frequency (HIDF) curve for Chicago including Gaussian perturbations along the duration axis, with a standard deviation of 0.05. This figure shows the frequency in terms of exceedance probability rather than non-exceedance probability. The red values on the left axis represent exceedance probabilities.


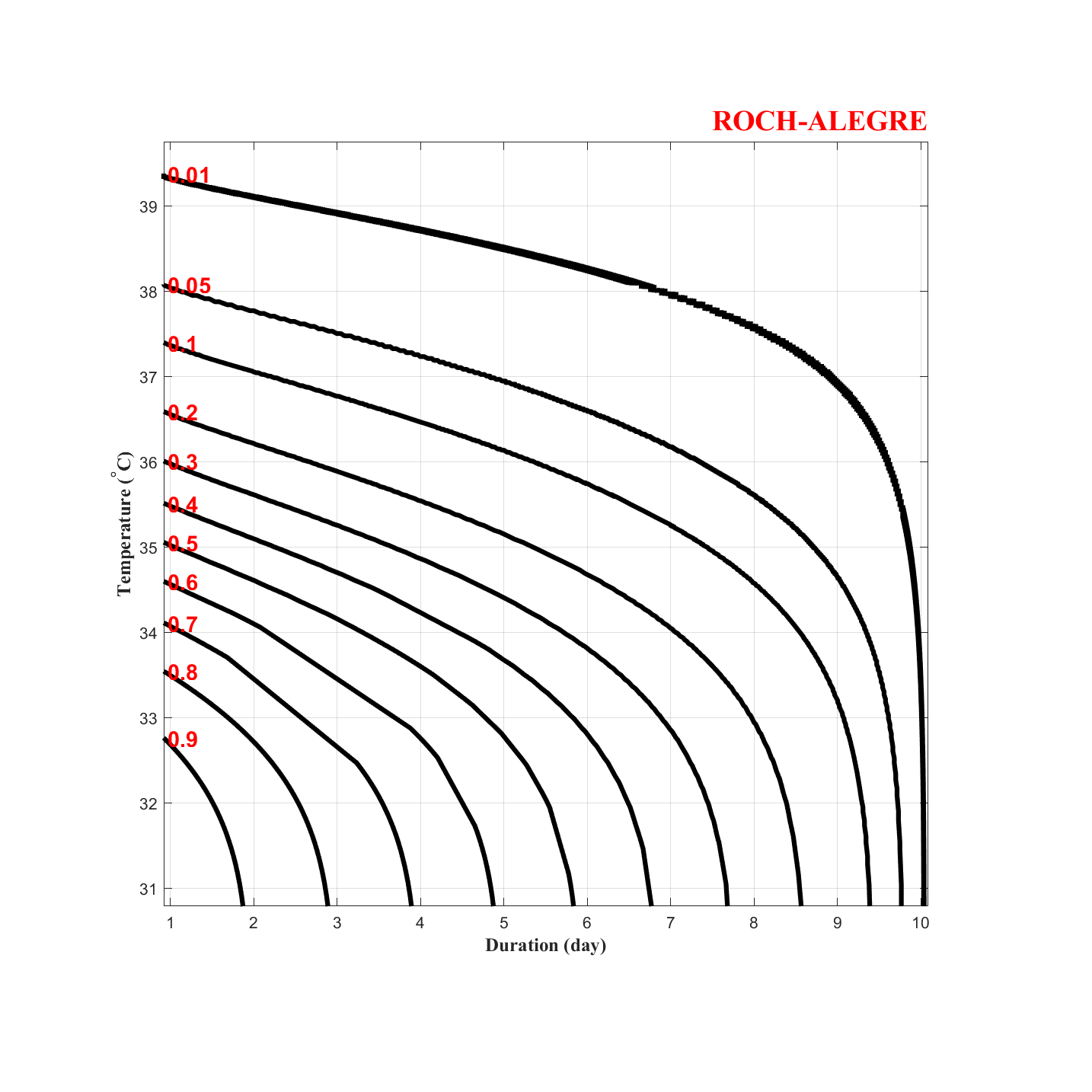


Figure S14: Heat wave intensity-duration-frequency (HIDF) curve for Denver including Gaussian perturbations along the duration axis, with a standard deviation of 0.05. This figure shows the frequency in terms of exceedance probability rather than non-exceedance probability. The red values on the left axis represent exceedance probabilities.


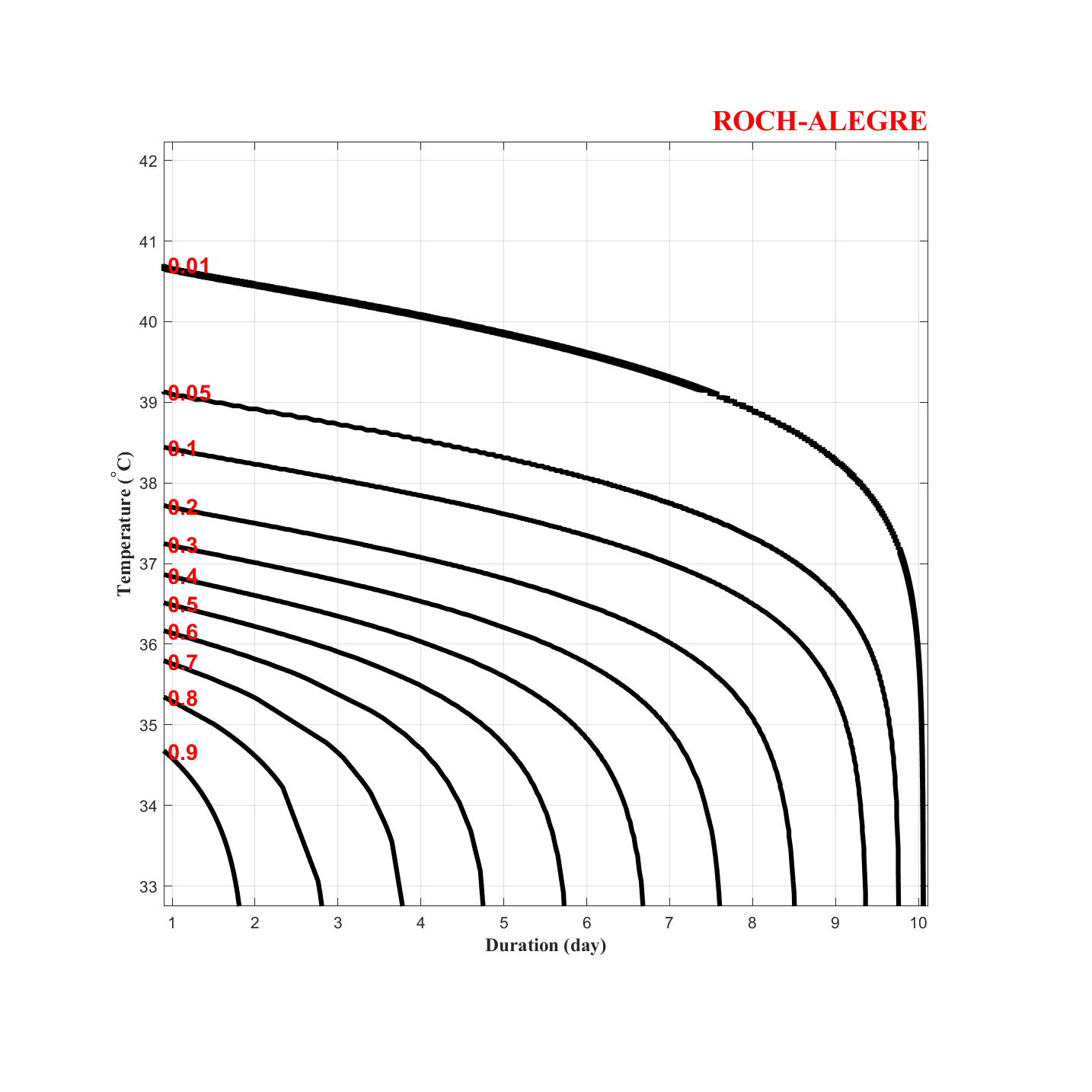


Figure S15: Heat wave intensity-duration-frequency (HIDF) curve for Houston including Gaussian perturbations along the duration axis, with a standard deviation of 0.05. This figure shows the frequency in terms of exceedance probability rather than non-exceedance probability. The red values on the left axis represent exceedance probabilities.


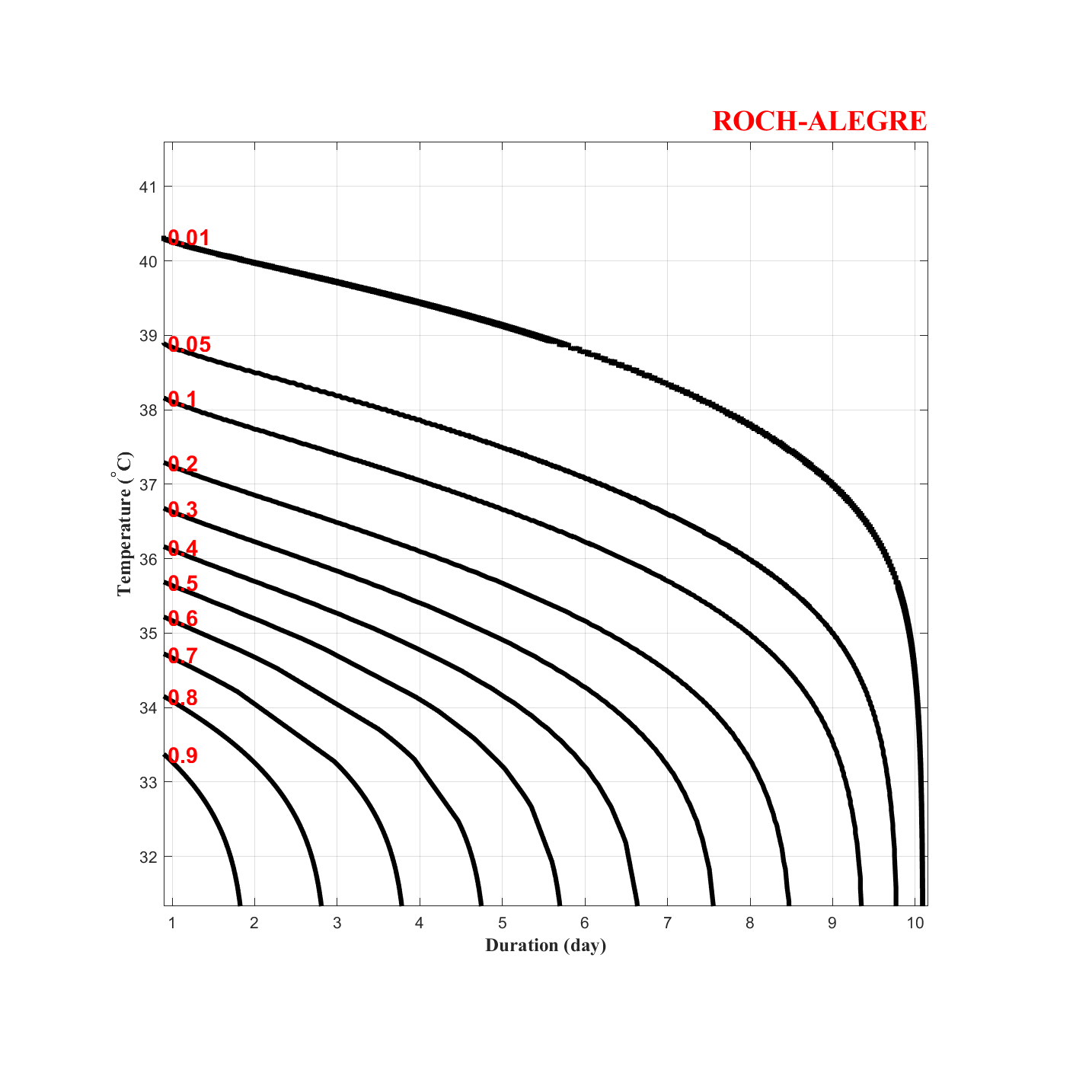


Figure S16: Heat wave intensity-duration-frequency (HIDF) curve for Los Angeles including Gaussian perturbations along the duration axis, with a standard deviation of 0.05. This figure shows the frequency in terms of exceedance probability rather than non-exceedance probability. The red values on the left axis represent exceedance probabilities.


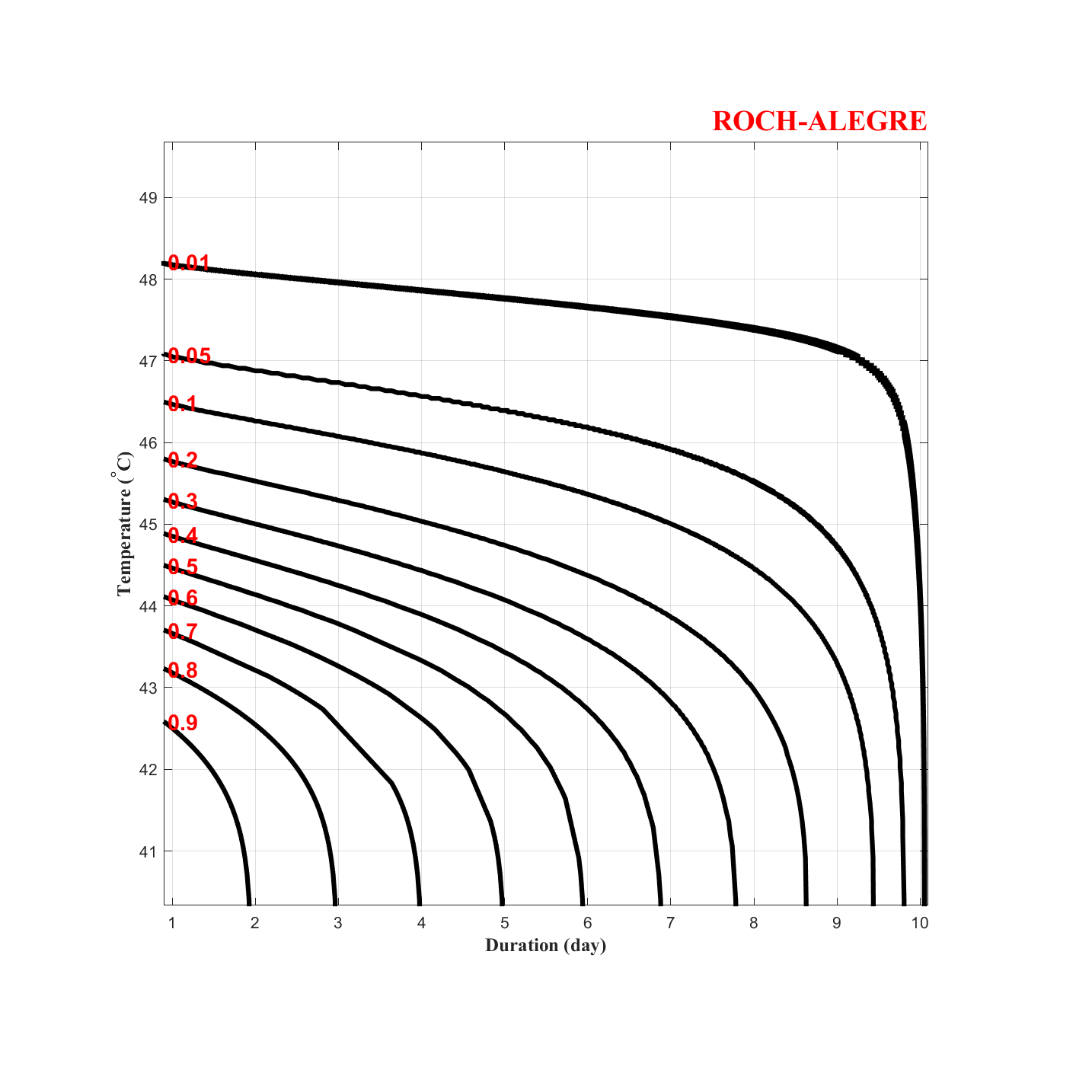


Figure S17: Heat wave intensity-duration-frequency (HIDF) curve for Phoenix including Gaussian perturbations along the duration axis, with a standard deviation of 0.05. This figure shows the frequency in terms of exceedance probability rather than non-exceedance probability. The red values on the left axis represent exceedance probabilities.


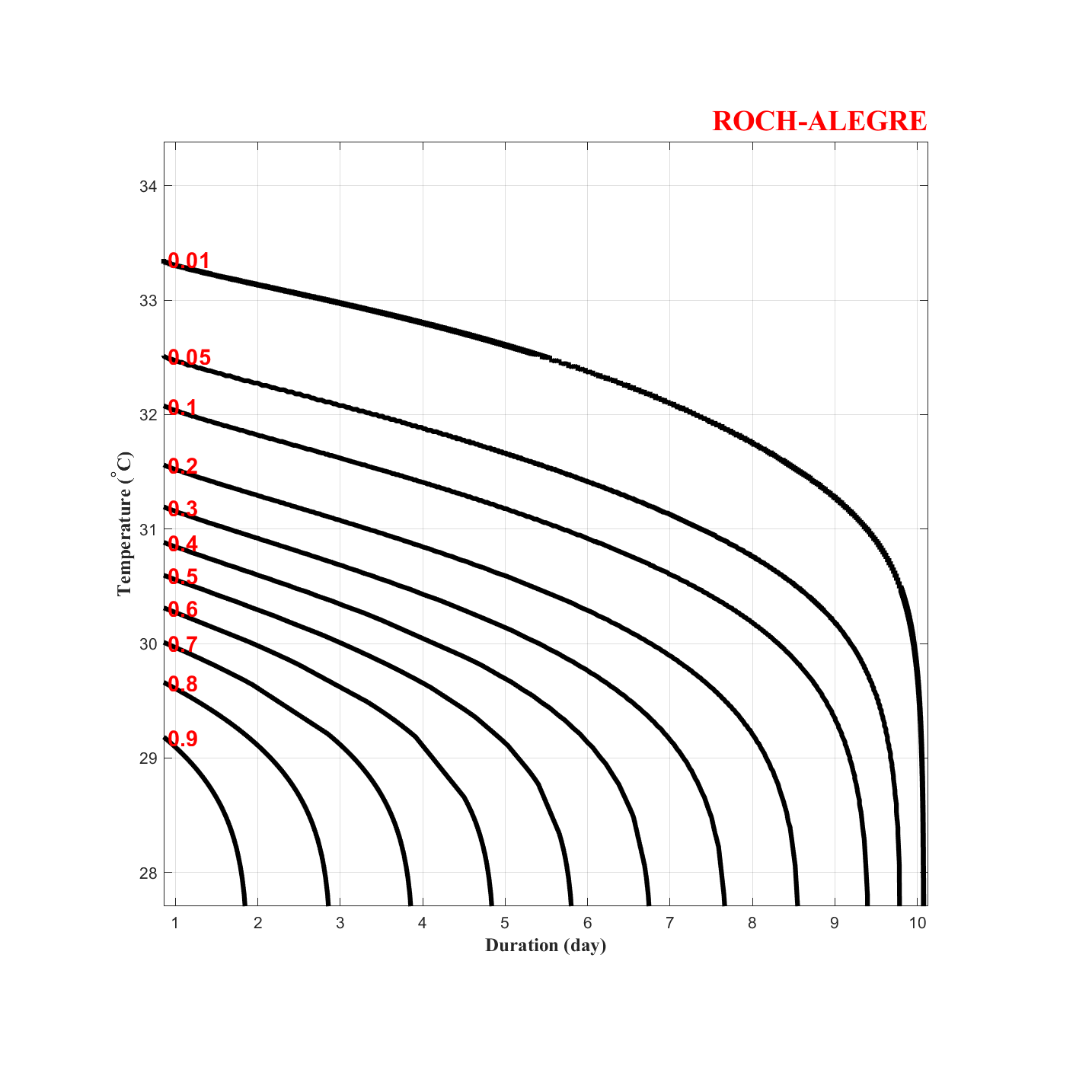


Figure S18: Mean heat wave intensity-duration-frequency (HIDF) curve for historical CMIP 5 simulations including Gaussian perturbations along the duration axis, with a standard deviation of 0.05. This figure shows the frequency in terms of exceedance probability rather than non-exceedance probability. The red values on the left axis represent exceedance probabilities.


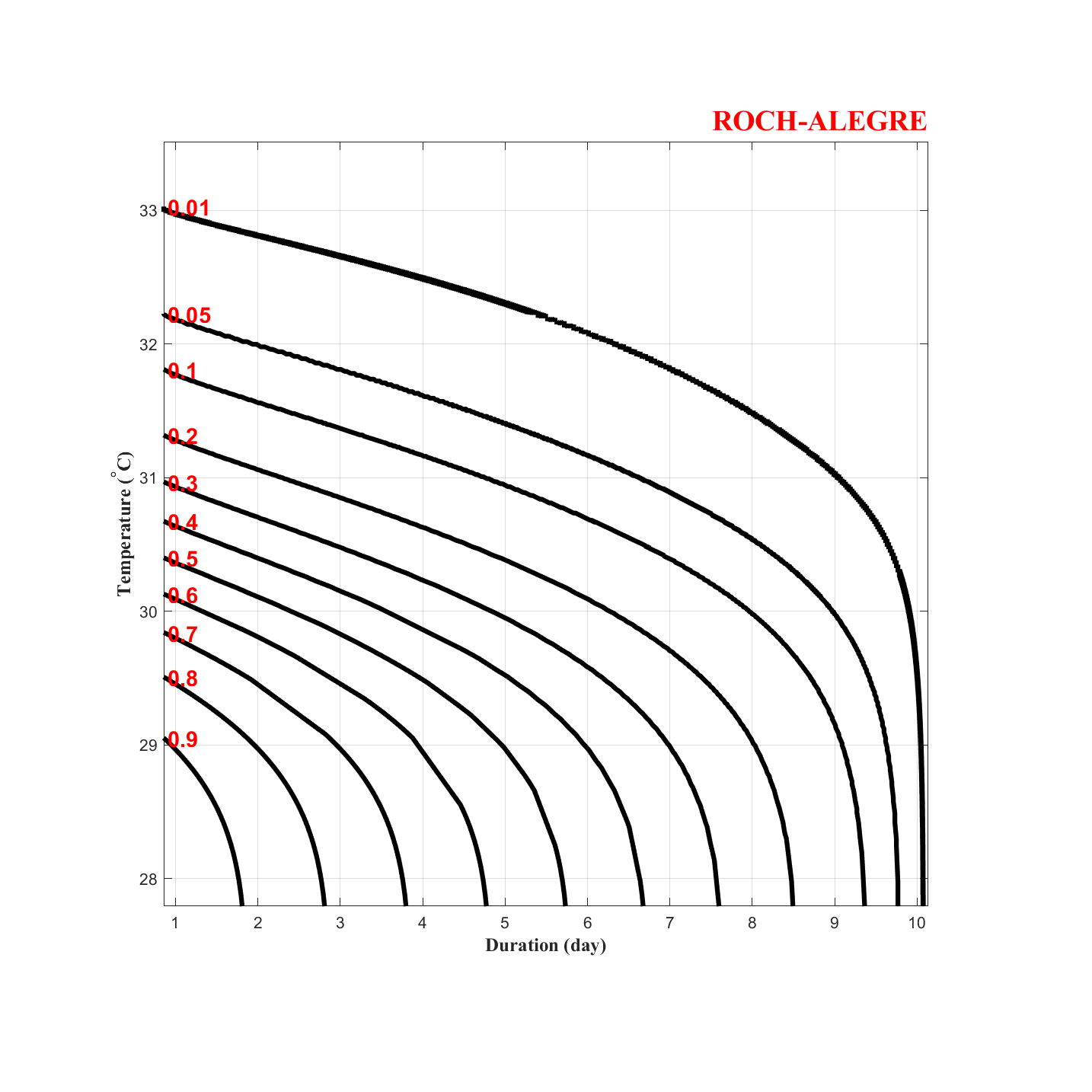


Figure S19: Mean heat wave intensity-duration-frequency (HIDF) curve for natural-only historical CMIP 5 simulations including Gaussian perturbations along the duration axis, with a standard deviation of 0.05. This figure shows the frequency in terms of exceedance probability rather than non-exceedance probability. The red values on the left axis represent exceedance probabilities.


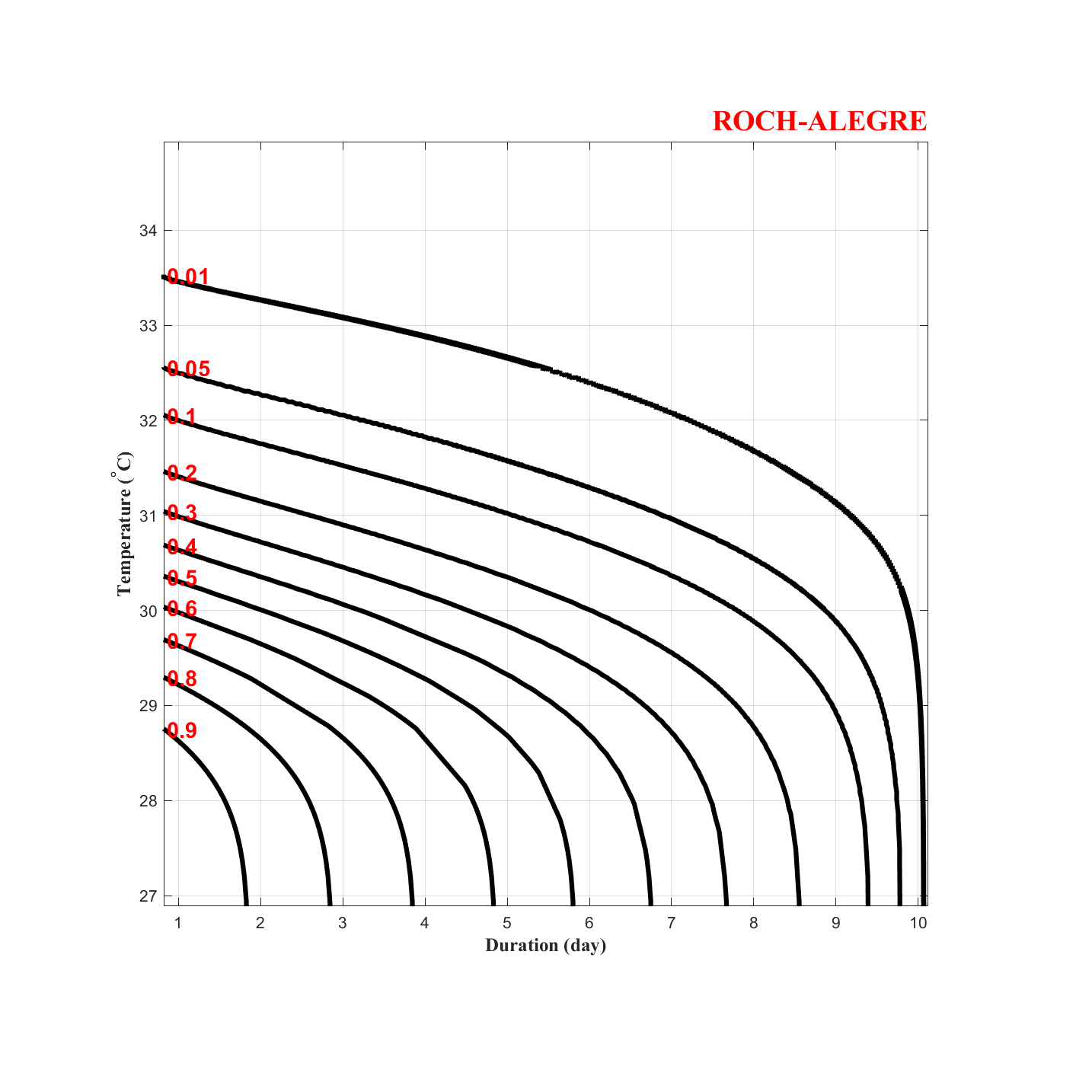


Figure S20: Median heat wave intensity-duration-frequency (HIDF) curve for historical CMIP 5 simulations including Gaussian perturbations along the duration axis, with a standard deviation of 0.05. This figure shows the frequency in terms of exceedance probability rather than non-exceedance probability. The red values on the left axis represent exceedance probabilities.


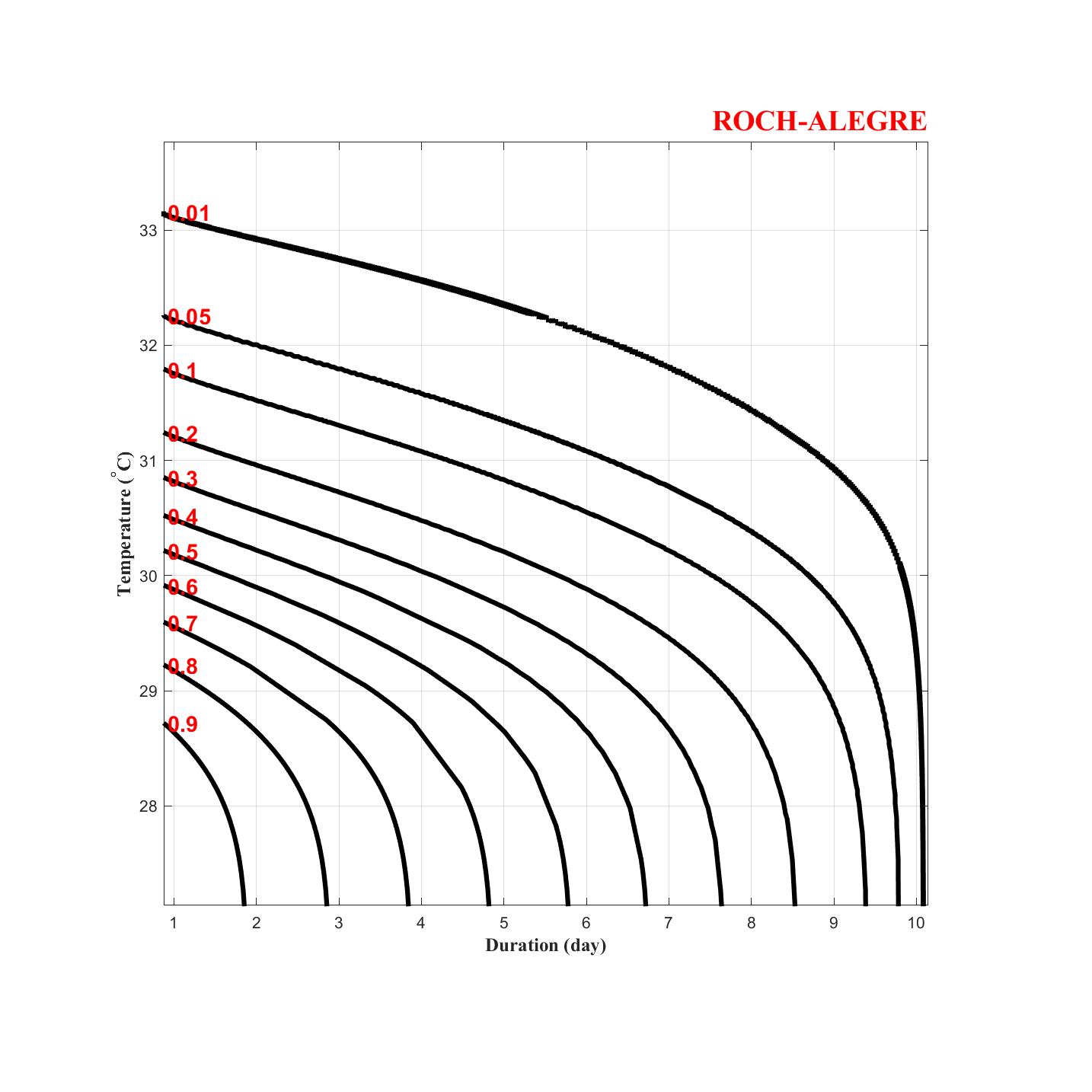


Figure S21: Median heat wave intensity-duration-frequency (HIDF) curve for natural-only historical CMIP 5 simulations including Gaussian perturbations along the duration axis, with a standard deviation of 0.05. This figure shows the frequency in terms of exceedance probability rather than non-exceedance probability. The red values on the left axis represent exceedance probabilities.


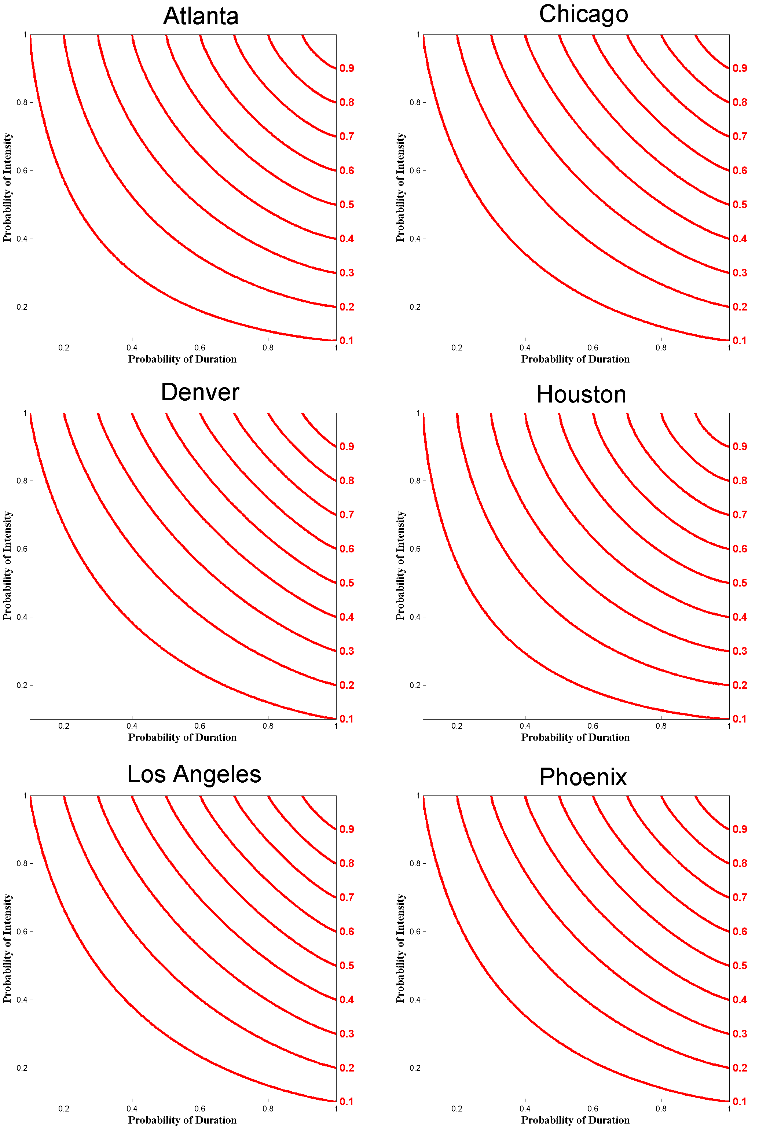


Figure 22: This figure shows the probability space copulas of heat wave duration and intensity. The red values on the right axis represent non-exceedance probabilities.

Figure 23: Normalized rank and empirical non-exceedance probabilities for Atlanta.

Figure 24: Normalized rank and empirical non-exceedance probabilities for Chicago.

Figure 25: Normalized rank and empirical non-exceedance probabilities for Denver.

Figure 26: Normalized rank and empirical non-exceedance probabilities for Houston.

Figure 27: Normalized rank and empirical non-exceedance probabilities for Los Angeles.

Figure 28: Normalized rank and empirical non-exceedance probabilities for Phoenix.


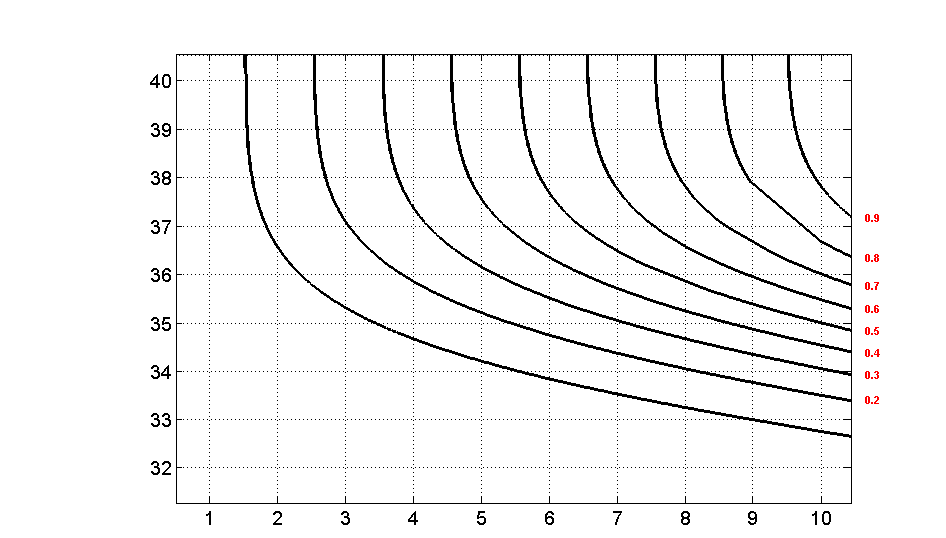


Figure S29: Heat wave intensity-duration-frequency (HIDF) curve for Atlanta including uniform perturbations along the duration axis, over (-0.5,0.5). The red values on the right axis represent non-exceedance probabilities.


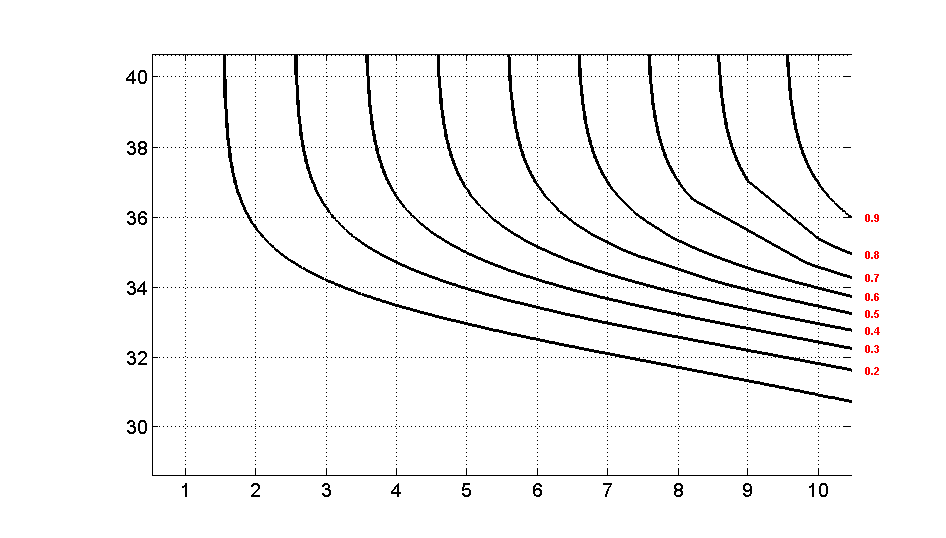


Figure S30: Heat wave intensity-duration-frequency (HIDF) curve for Chicago including uniform perturbations along the duration axis, over (-0.5,0.5). The red values on the right axis represent non-exceedance probabilities.


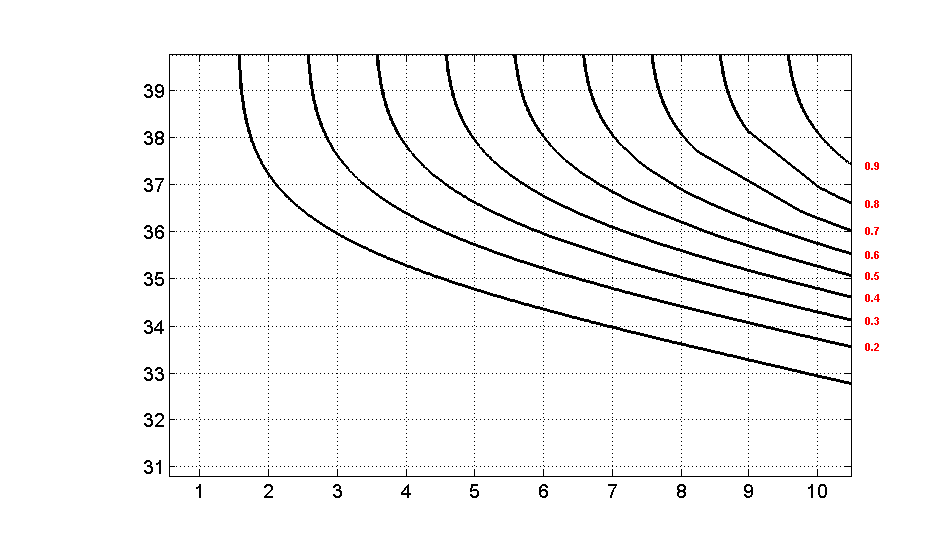


Figure S31: Heat wave intensity-duration-frequency (HIDF) curve for Denver including uniform perturbations along the duration axis, over (-0.5,0.5). The red values on the right axis represent non-exceedance probabilities.


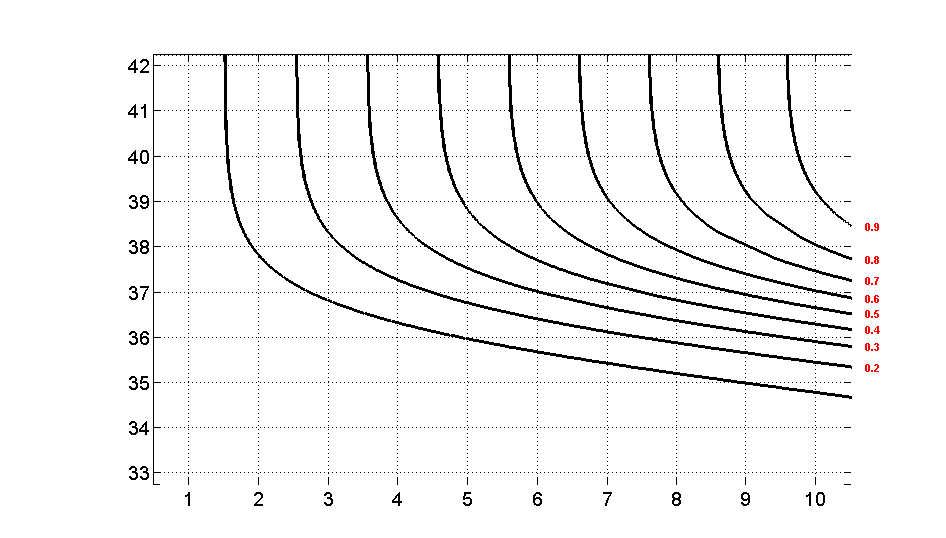


Figure S32: Heat wave intensity-duration-frequency (HIDF) curve for Houston including uniform perturbations along the duration axis, over (-0.5,0.5). The red values on the right axis represent non-exceedance probabilities.


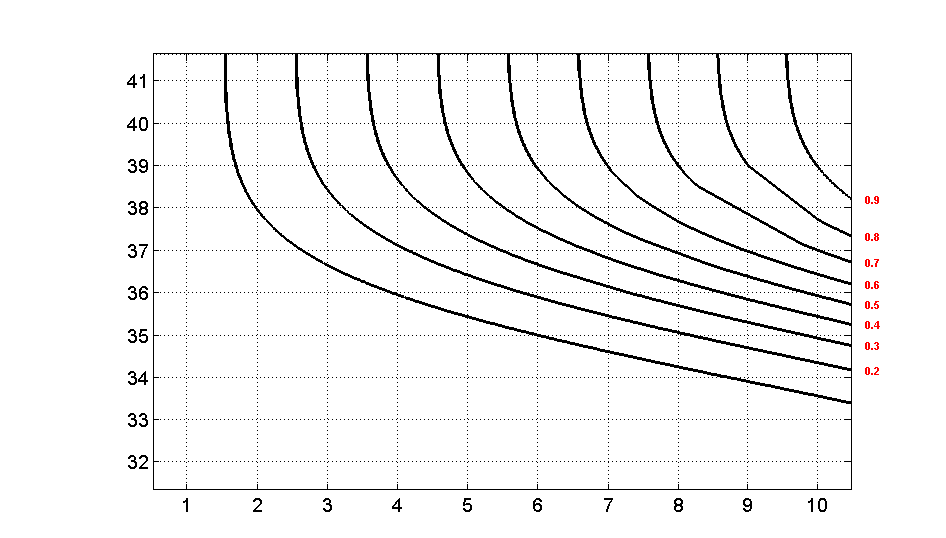


Figure S33: Heat wave intensity-duration-frequency (HIDF) curve for Los Angeles including uniform perturbations along the duration axis, over (-0.5,0.5). The red values on the right axis represent non-exceedance probabilities.


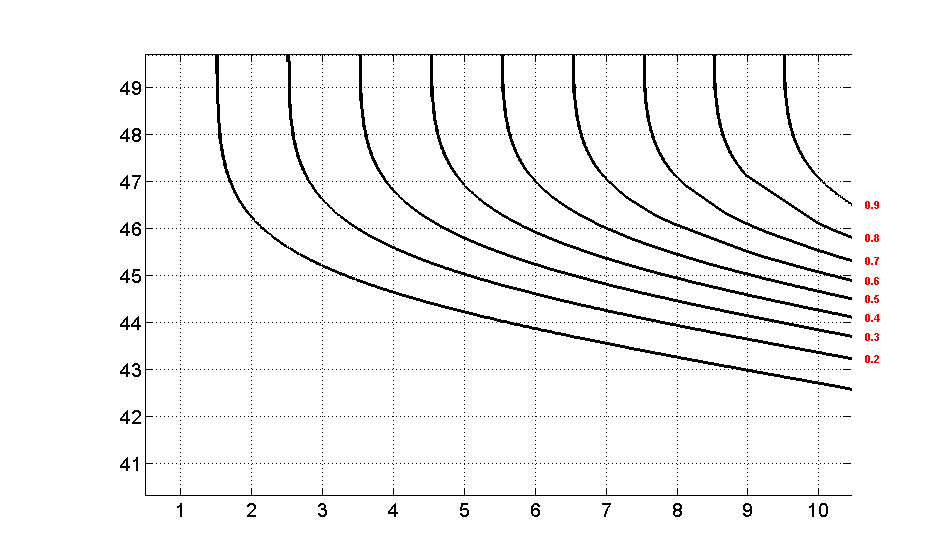


Figure S34: Heat wave intensity-duration-frequency (HIDF) curve for Phoenix including uniform perturbations along the duration axis, over (-0.5,0.5). The red values on the right axis represent non-exceedance probabilities.


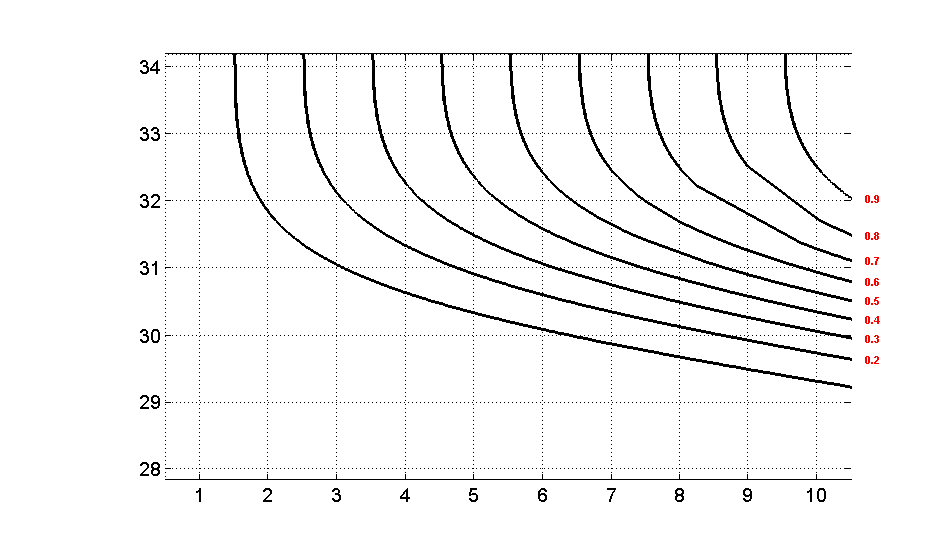


Figure S35: Heat wave intensity-duration-frequency (HIDF) curve for mean historical CMIP5 simulations including uniform perturbations along the duration axis, over (-0.5,0.5). The red values on the right axis represent non-exceedance probabilities.


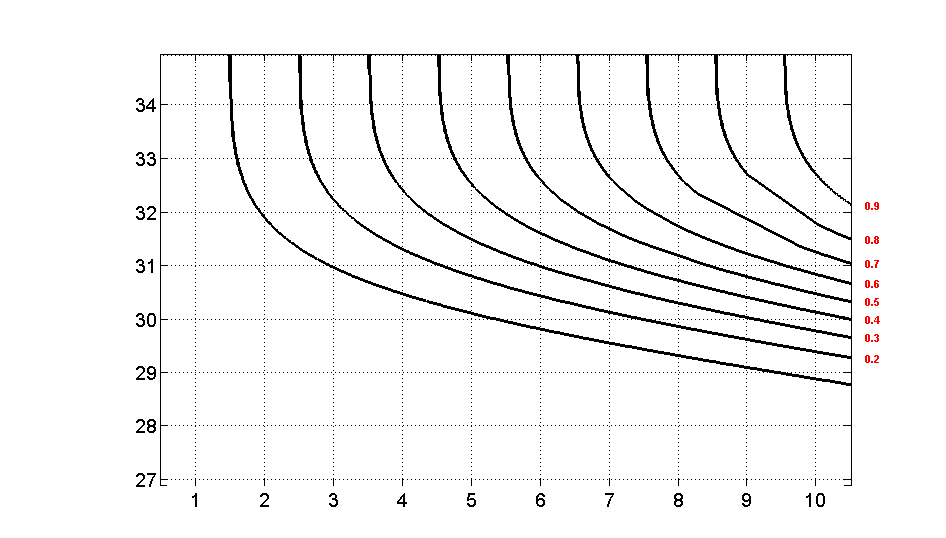


Figure S36: Heat wave intensity-duration-frequency (HIDF) curve for median historical CMIP5 simulations including uniform perturbations along the duration axis, over (-0.5,0.5). The red values on the right axis represent non-exceedance probabilities.


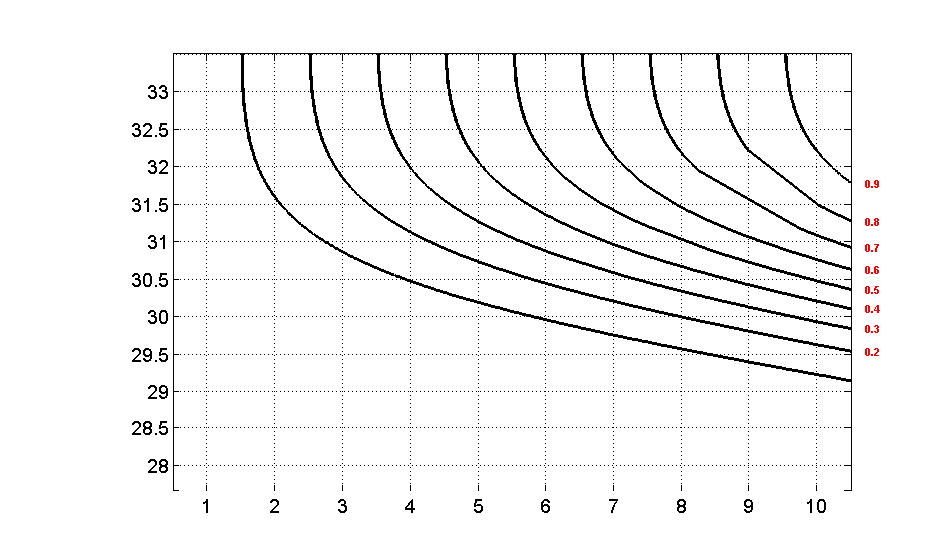


Figure S37: Heat wave intensity-duration-frequency (HIDF) curve for mean natural-only historical CMIP5 simulations including uniform perturbations along the duration axis, over (-0.5,0.5). The red values on the right axis represent non-exceedance probabilities.


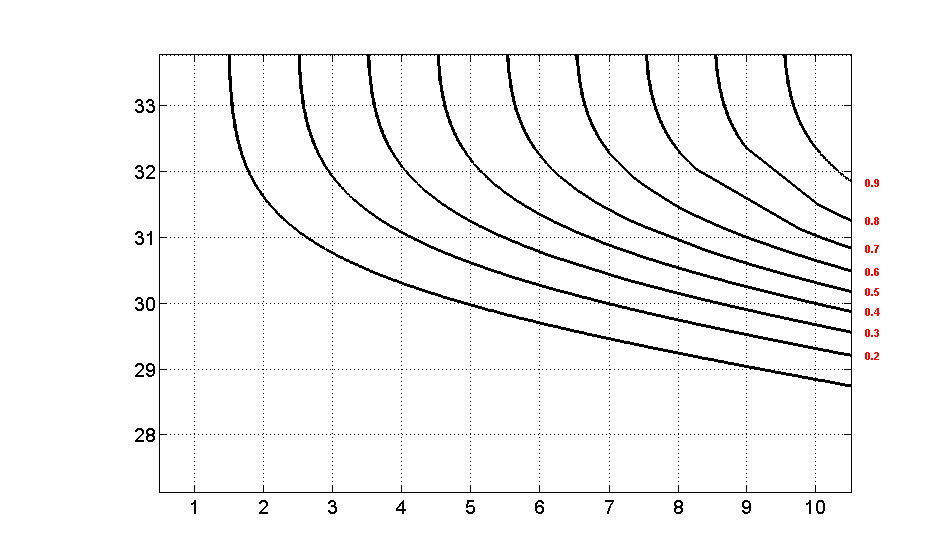


Figure S38: Heat wave intensity-duration-frequency (HIDF) curve for median natural-only historical CMIP5 simulations including uniform perturbations along the duration axis, over (-0.5,0.5). The red values on the right axis represent non-exceedance probabilities.

Bootstrap hypothesis test to evaluate difference between intensity of ten-year, ten-day heat wave event in historical and historical natural-only simulations:

We conducted a bootstrap hypothesis test to evaluate the significance of the difference between heat wave intensities simulated in historical and historical natural-only conditions. As noted in the main manuscript, we found a 0.3 ^o^C difference between the ten-year ten-day historical and historical natural-only heat wave intensities in Los Angeles, CA (Figure 3). To conduct this bootstrap test, we resampled (with replacement) 1560 pairs of heat wave intensity and duration from the block maxima temperature values and their corresponding durations from the historical natural-only model average to match the original length of the dataset. Then, we found the resampled historical natural-only heat wave intensity associated with the ten-year ten-day event. With 500 iterations, the 0.3 ^o^C difference between the historical and historical natural-only intensities was never equaled or exceeded, which shows that the difference is statistically significant (p-value < 0.01).
