# Supplementary material for: Heat wave Intensity Duration Frequency Curve: A Multivariate Approach for Hazard and Attribution Analysis
Source: Sci Rep. 2019 Oct 1;9:14117. doi: 10.1038/s41598-019-50643-w (PMC6773721; doi:10.1038/s41598-019-50643-w)
Supplement: Supplementary file 2 — Supplementary Tables [file 41598_2019_50643_MOESM2_ESM.docx]

**Supplementary Information – Tables**

**Heat wave Intensity Duration Frequency Curve: A Multivariate Approach for Hazard and Attribution Analysis**

Omid Mazdiyasni^1*^, Mojtaba Sadegh^2^, Felicia Chiang^1^, Amir AghaKouchak^1, 3^

^1^ Department of Civil and Environmental Engineering, University of California, Irvine, California 92697, USA

^2^ Department of Civil and Environmental Engineering, Boise State University, Idaho 83725, USA

^3^ Department of Earth System Science, University of California, Irvine, California 92697, USA

Table S1: List of climate models, their spatial resolutions, and Modeling Centers.

| Modeling Center | Institute ID | Model Name | Spatial resolution (lat, lon) |
| --- | --- | --- | --- |
| National Center for Atmospheric Research | NCAR | CCSM4 | 0.9424, 1.25 |
| Commonwealth Scientific and Industrial Research Organization in collaboration with Queensland Climate Change Centre of Excellence | CSIRO-QCCCE | CSIRO-Mk3.6.0 | 1.8653, 1.875 |
| Japan Agency for Marine-Earth Science and Technology, Atmosphere and Ocean Research Institute (The University of Tokyo), and National Institute for Environmental Studies | MIROC | MIROC-ESM | 2.7906, 2.8125 |
| Norwegian Climate Centre | NCC | NorESM1-M | 1.8947, 2.5 |

# Marginal distributions:

We use 17 different continuous marginal distribution functions to find a suitable model that optimally fits the available data. Distribution functions include (1) Beta, (2) Birnbaum-Saunders, (3) exponential, (4) extreme value, (5) Gamma, (6) generalized extreme value, (7) generalized Pareto, (8) inverse Gaussian, (9) logistic, (10) log-logistic, (11) lognormal, (12) Nakagami, (13) normal, (14) Rayleigh, (15) Rician, (16) t location scale, and (17) Weibull distributions (listed alphabetically).

Table S2: Summary of chosen marginal distributions and copula families. The marginal distributions were chosen by the Chi-Square goodness of fit test, with a significance level of 0.05. The copula family was chosen based on the p-value, maximum likelihood, Akaike Information Criterion (AIC), and Bayesian Information Criterion (BIC). RMSE stands for Root Mean Square Error, NSE stands for Nash-Sutcliffe efficiency, GP stands for Generalized Pareto Distribution, GEV stands for Generalized Extreme Value Distribution. The null hypothesis is that the underlying copula is a member of the parametric copula. If the p-value is below 0.05, the null hypothesis is rejected, and if the p-value is greater than 0.05, the null hypothesis cannot be rejected ^50,51^.

| Dataset | Duration Marginal Distribution | Intensity Marginal Distribution | Copula Family | COPULA  RMSE | COPULA  NSE | COPULA p-value |
| --- | --- | --- | --- | --- | --- | --- |
| Atlanta | GP | Inverse Gaussian | Roche-Alegre | 0.3798 | 0.9887 | 0.51 |
| Chicago | GP | Loglogistic | Roche-Alegre | 0.3830 | 0.9847 | 0.90 |
| Denver | GP | Nakagami | Roche-Alegre | 0.4062 | 0.9838 | 0.90 |
| Houston | GP | Loglogistic | Roche-Alegre | 0.4052 | 0.9869 | 0.54 |
| Los Angeles | GP | Lognormal | Roche-Alegre | 0.4433 | 0.9784 | 0.65 |
| Phoenix | GP | Lognormal | Roche-Alegre | 0.3999 | 0.9843 | 0.66 |
| Historical | GP | GEV | Roche-Alegre | 0.8932 | 0.9620 | 0.52 |
| Natural-Only Historical | GP | GEV | Roche-Alegre | 0.9967 | 0.9424 | 0.48 |
